# Basal ganglia–spinal cord pathway that commands locomotor gait asymmetries in mice

**DOI:** 10.1038/s41593-024-01569-8

**Published:** 2024-02-12

**Authors:** Jared M. Cregg, Simrandeep K. Sidhu, Roberto Leiras, Ole Kiehn

**Affiliations:** 1 Department of Neuroscience, Faculty of Health and Medical Sciences, University of Copenhagen, Copenhagen, Denmark; 2 Department of Neuroscience, Karolinska Institutet, Stockholm, Sweden

**Keywords:** Basal ganglia, Spinal cord

## Abstract

The basal ganglia are essential for executing motor actions. How the basal ganglia engage spinal motor networks has remained elusive. Medullary *Chx10* gigantocellular (Gi) neurons are required for turning gait programs, suggesting that turning gaits organized by the basal ganglia are executed via this descending pathway. Performing deep brainstem recordings of *Chx10* Gi Ca^2+^ activity in adult mice, we show that striatal projection neurons initiate turning gaits via a dominant crossed pathway to *Chx10* Gi neurons on the contralateral side. Using intersectional viral tracing and cell-type-specific modulation, we uncover the principal basal ganglia–spinal cord pathway for locomotor asymmetries in mice: basal ganglia → pontine reticular nucleus, oral part (PnO) → *Chx10* Gi → spinal cord. Modulating the restricted PnO → *Chx10* Gi pathway restores turning competence upon striatal damage, suggesting that dysfunction of this pathway may contribute to debilitating turning deficits observed in Parkinson’s disease. Our results reveal the stratified circuit architecture underlying a critical motor program.

## Abstract (web summary)

The basal ganglia control the execution of motor actions. However, how they engage spinal motor networks is unclear. Here the authors show that the basal ganglia–spinal cord pathway controls locomotor asymmetries in adult mice.

## Main

The basal ganglia are essential for motor action commitment. Basal ganglia control of motor actions has traditionally been examined in the context of cortico–striatal–thalamocortical loops. Nonetheless, the brainstem represents a major target of basal ganglia output^1,2^. Within the brainstem, diverse motor programs organize specific actions, including visual saccades, head direction, reach/grasp, orofacial movements and locomotion^3–7^. Previous work has utilized unitary recordings, electrical stimulation, optogenetic manipulations and/or pharmacology^8–13^ to indicate that basal ganglia control over motor programs operates via nigral disinhibition of target structures^14–18^. Nevertheless, it remains crucial to delineate how the basal ganglia interface with specific brainstem motor pathways, and to identify the distinct circuit motifs that facilitate execution of motor actions at the spinal level^5,19^.

The basal ganglia are especially critical for locomotion^3,5,6^. Locomotion requires precise rhythm and coordination which arises largely due to network properties intrinsic to the spinal cord itself^6,20,21^. Recent data have revealed that distinct aspects of locomotor control are recruited via specific populations of brainstem reticulospinal neurons, including those responsible for locomotor initiation, speed, stop and turn^6,22–28^. In particular, these data show that excitatory *Chx10* Gi reticulospinal projection neurons in the medulla are required for turning gait asymmetries^23^. The primacy of locomotion is exemplified in parkinsonian patients, which exhibit a number of locomotor abnormalities including bradykinesia, freezing of gait and exacerbated turning deficits^29–33^. Turning gait deficits are especially prominent in advanced stages of Parkinson’s disease, representing a defining feature of parkinsonian gait^34^. Turning in Parkinson’s disease is characterized by increased turning duration, an increased number of small steps to complete a turn and impaired rotational coordination^30,31,35,36^.

Using Ca^2+^ recording, intersectional viral tracing, and gain- and loss-of-function optogenetic experiments in freely moving mice, we reveal that a pontine reticular nucleus, oral part (PnO) → *Chx10* Gi → spinal cord pathway is largely responsible for basal ganglia-induced turns. Furthermore, we used this specific pathway information to demonstrate the possibility of restoring turning competence in an experimental parkinsonian mouse model. These data provide a direct circuit-level explanation for basal ganglia-induced turns, and suggest a role of these circuits in turning deficits observed in basal ganglia disorders.

## Results

### Brainstem *Chx10* Gi neurons encode turning gait asymmetries

**Fig. 1:**
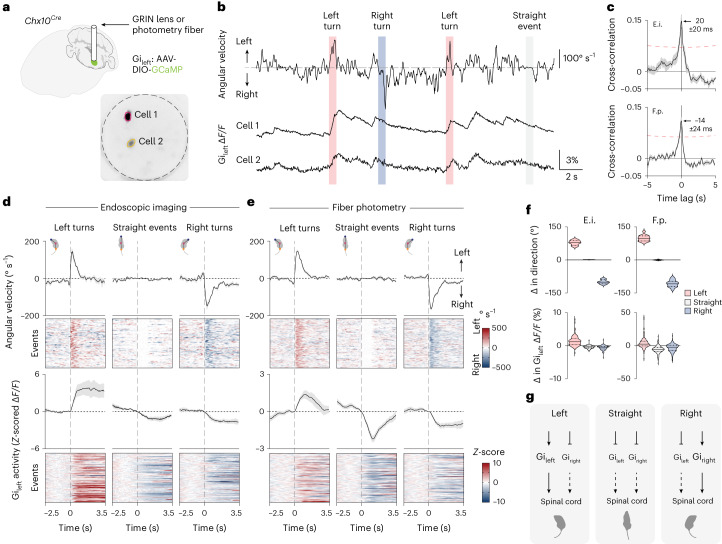
Brainstem *Chx10* Gi neurons encode turning gait asymmetries. **a**, Schematic of *Chx10* Gi_left_ GCaMP recording. Example endoscopic Ca^2+^ imaging field of view (inverted maximum Δ*F/F* projection). **b**, Example Δ*F/F* traces from cells in **a** with concurrent tracking of body angular velocity. **c**, Top, cross-correlation between body angular velocity and the derivative of *Chx10* Gi_left_ Δ*F/F* activity obtained from endoscopic imaging (E.i.; *n* = 5 neurons from 4 mice from 3 independent experiments). Bottom, cross-correlation between body angular velocity and the derivative of *Chx10* Gi_left_ Δ*F/F* activity obtained using fiber photometry (F.p.; *n* = 14 mice from 4 independent experiments). A negative time lag indicates that the derivative of the Δ*F/F* signal leads the angular velocity signal. Dashed red lines represent bounds for significant correlation. **d**,**e**, Spontaneous left turns, straight events or right turns were segmented from angular velocity time series. Left turns were associated with an increase in *Chx10* Gi_left_ activity, whereas straight events and right turns were associated with a decrease in *Chx10* Gi_left_ activity. Endoscopic imaging: *n* = 5 neurons from 4 mice, 20 left turns, 20 straight events and 20 right turns for each mouse (**d**). Fiber photometry: *n* = 14 mice, 10 left turns, 10 straight events and 10 right turns for each mouse (**e**). Red denotes a positive angular velocity (left turn) or *Z*-score. Blue signifies a negative angular velocity (right turn) or *Z*-score. Error bands in **c**–**e** represent the s.e.m. **f**, Top, change in direction associated with spontaneous left turns, straight events or right turns segmented from angular velocity time series. Bottom, change in *Chx10* Gi_left_ Δ*F/F* activity associated with spontaneous left turns, straight events or right turns. Endoscopic imaging: *n* = 5 neurons from 4 mice from 3 independent experiments. Fiber photometry: *n* = 14 mice from 4 independent experiments. Violin plots give the median, the 25th and 75th percentiles and the range. See Supplementary Table 1 for full statistical analysis. **g**, Model for *Chx10* Gi activity during spontaneous changes in locomotor direction. Spontaneous left turns are associated with both an increase in *Chx10* Gi_left_ activity and a decrease in *Chx10* Gi_right_ activity.

Medullary *Chx10*-lineage Gi (*Chx10* Gi) neurons represent a reticulospinal command line required for turning^23^. Unilateral activation of *Chx10* Gi neurons causes turning toward the ipsilateral side, whereas unilateral inhibition causes turning toward the contralateral side^23,28^. *Chx10* Gi neurons are located dorsomedial to the lateral paragigantocellular nucleus^37^, where a separate population of reticulospinal neurons has been implicated in locomotor initiation and speed^27,38^. To investigate how *Chx10* Gi neurons encode turning, we performed deep brainstem GCaMP Ca^2+^ recording of *Chx10* Gi neurons in freely moving mice using endoscopic imaging and fiber photometry (Fig. 1a).

Endoscopic Ca^2+^ imaging revealed that single *Chx10* Gi neurons encode turning velocity (Fig. 1a–d and Supplementary Video 1). Of seven cells obtained from five mice, five cells exhibited a significant cross-correlation between body angular velocity and the derivative of the change in Ca^2+^ fluorescence (Δ*F/F*) (Fig. 1c). Rise in Ca^2+^ activity preceded turn onset, where cross-correlation surpassed the threshold for statistical significance (Fig. 1c) at −400 ± 115 ms and exhibited a peak correlation at 20 ± 20 ms (Fig. 1c). We segmented spontaneous left turns, straight events and right turns from angular velocity profiles (Fig. 1d and Supplementary Video 2). Spontaneous left turns (peak angular velocity greater than 200° s^−1^) were correlated with an increase in Δ*F/F* activity of cells recorded in Gi_left_, whereas right turns (less than −200° s^−1^) were associated with a decrease in Gi_left_ Δ*F/F* activity (Fig. 1d,f and Supplementary Table 1). Straight events (±20° s^−1^) were also correlated with a decrease in Gi_left_ Δ*F/F* activity.

Fiber photometry revealed a direct correspondence between activity of single *Chx10* Gi neurons and activity at the population level. *Chx10* Gi_left_ population Ca^2+^ activity correlated with spontaneous leftward movements and was anti-correlated with rightward movements (Fig. 1c,e). In 14 of 14 mice, the rise in Ca^2+^ activity preceded turn onset, with a significant cross-correlation between body angular velocity and the derivative of Δ*F/F* at −240 ± 53 ms and a peak correlation at −14 ± 24 ms (Fig. 1c). Furthermore, spontaneous left turns segmented from angular velocity profiles were correlated with an increase in *Chx10* Gi_left_ population Ca^2+^ activity, whereas right turns were associated with a decrease in Ca^2+^ population activity (Fig. 1e,f, Supplementary Video 2 and Supplementary Table 1). Similar to single cell recordings, straight events were also correlated with a decrease in Ca^2+^ population activity (Fig. 1e,f). These data indicate that *Chx10* Gi population activity encodes body angular velocity. Spontaneous turns are associated with bilateral modulation of *Chx10* Gi activity: an increase in activity on the side of the turn and a decrease in activity on the contralateral side (Fig. 1f,g), consistent with unilateral *Chx10* Gi gain- and loss-of-function experiments^23,28^.

### *Chx10* Gi neurons encode striatal turning gait asymmetries

**Fig. 2:**
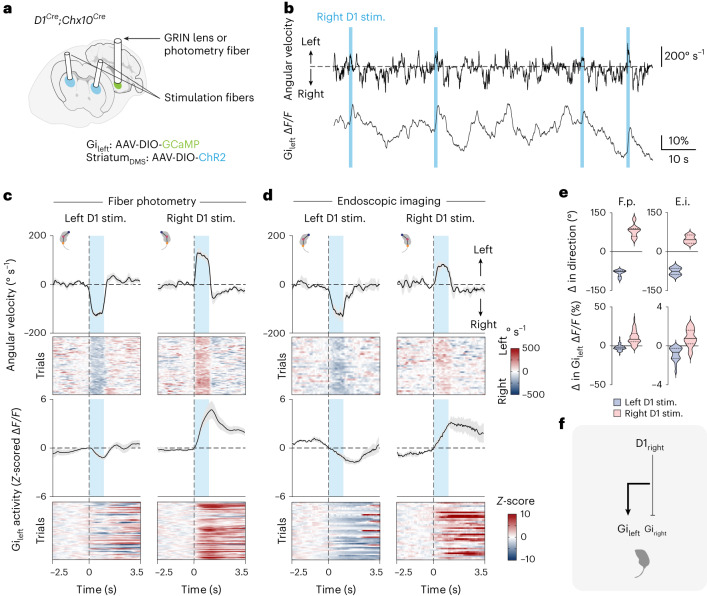
Stereotypic modulation of *Chx10* Gi activity via unilateral activation of D1 SPNs. **a**, Schematic of *Chx10* Gi_left_ GCaMP recording with optogenetic stimulation of left or right D1 SPNs. **b**, Example fiber photometry trace with concurrent tracking of body angular velocity. *Chx10* Gi_left_ activity correlated with left turns, and was anti-correlated with right turns. Stimulation of right D1 SPNs evoked left turns accompanied by an increase in *Chx10* Gi_left_ activity. **c**,**d**, Stimulation of D1 SPNs evoked contraversive turns, with an increase in *Chx10* Gi activity contralateral to the stimulation and decrease in *Chx10* Gi activity ipsilateral to the stimulation. Fiber photometry: *n* = 7 mice from 2 independent experiments; 10 left and right D1 stimulation trials for each mouse (**c**). Endoscopic imaging: Left D1 stim.: *n* = 3 cells from 2 mice in 2 independent experiments; 12–20 trials for each mouse. Right D1 stim.: *n* = 3 cells from 3 mice in 3 independent experiments; 7–20 trials for each mouse (**d**). Error bands in **c** and **d** represent the s.e.m. **e**, Top, change in direction associated with 1-s ChR2 stimulation of left or right D1 SPNs. Bottom, change in *Chx10* Gi_left_ Δ*F/F* activity associated with 1-s ChR2 stimulation of left or right D1 SPNs. The magnitude of the change in Δ*F/F* was greater for D1_right_ versus D1_left_ stimulation trials; *P = 0.037; two-tailed paired *t*-test; *n* = 7 mice from 2 independent experiments. Violin plots give the median, the 25th and 75th percentiles and the range. See Supplementary Table 1 for full statistical analysis. **f**, Model for locomotor asymmetries caused by stimulation of D1 SPNs. Optogenetic stimulation of D1 SPNs has a dominant contralateral excitatory effect on *Chx10* Gi neurons, as well as a weaker ipsilateral inhibitory effect. Stim., stimulation.

**Extended Data Fig. 1:**
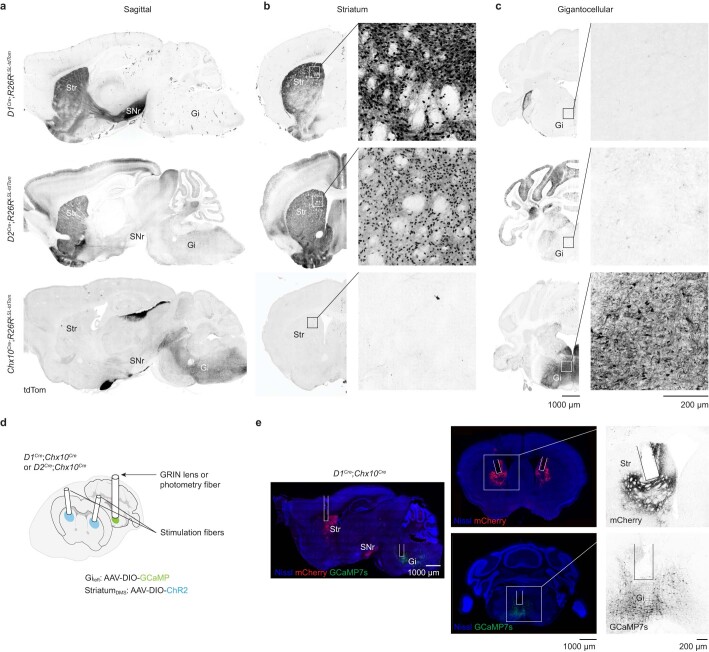
Optogenetic stimulation of SPNs with simultaneous Ca^2+^ recording from *Chx10* Gi neurons. **a**, *D1*^*Cre*^ and *D2*^*Cre*^, and *Chx10*^*Cre*^ alleles exhibit non-overlapping Cre expression within the striatum (Str) and nucleus gigantocellularis (Gi), respectively, in sagittal sections. *Top*, *D1*^*Cre*^ (Gensat EY217);*R26R*^*LSL-tdTom*^ mice exhibit tdTom expression in Str, with tdTom^+^ axons projecting to SNr. tdTom expression is largely absent from Gi. *Middle*, *D2*^*Cre*^ (Gensat ER44);*R26R*^*LSL-tdTom*^ mice exhibit tdTom expression in Str, whereas tdTom expression is largely absent from Gi. *Bottom*, *Chx10*^*Cre*^;*R26R*^*LSL-tdTom*^ mice exhibit tdTom expression in Gi, whereas tdTom expression is largely absent in Str. Sagittal images are representative of expression patterns observed in *n* = 3 mice from one experiment for each genotype. **b**, In coronal sections including the striatum, *D1*^*Cre*^;*R26R*^*LSL-tdTom*^ and *D2*^*Cre*^;*R26R*^*LSL-tdTom*^ mice exhibit strong tdTom expression in Str, whereas *Chx10*^*Cre*^;*R26R*^*LSL-tdTom*^ mice exhibit scant tdTom expression in Str. **c**, In coronal sections including Gi, *D1*^*Cre*^; *R26R*^*LSL-tdTom*^ and *D2*^*Cre*^;*R26R*^*LSL-tdTom*^ mice exhibit scant tdTom expression in Gi. *D2*^*Cre*^;*R26R*^*LSL-tdTom*^ mice exhibit some tdTom expression in the neighboring IRt and PCRt. *Chx10*^*Cre*^;*R26R*^*LSL-tdTom*^ mice exhibit strong tdTom expression in Gi. Coronal images are representative of expression patterns observed in *n* = 3–6 mice from one experiment for each genotype. Scale bar (**a-c**) for overview sagittal and coronal images = 1.0 mm. Scale bar (**b, c**) for insets = 200 µm. **d**, Schematic of *Chx10* Gi_left_ GCaMP recording with optogenetic stimulation of left or right, D1 or D2 SPNs. **e**, Examples of bilateral ChR2-2A-mCherry expression and fiber implantation in the dorsomedial Str, and GCaMP7s expression and fiber implantation in Gi_left_ in *D1*^*Cre*^;*Chx10*^*Cre*^ dual-allelic mice. ChR2-2A-mCherry and GCaMP7s expression patterns are representative of *n* = 7 *D1*^*Cre*^;*Chx10*^*Cre*^ mice from two independent experiments. *D2*^*Cre*^; *Chx10*^*Cre*^ mice exhibited similar ChR2-2A-mCherry and GCaMP7s expression patterns, with notable absence of mCherry^+^ axons in SNr (*n* = 7 mice from two independent experiments).

**Extended Data Fig. 2:**
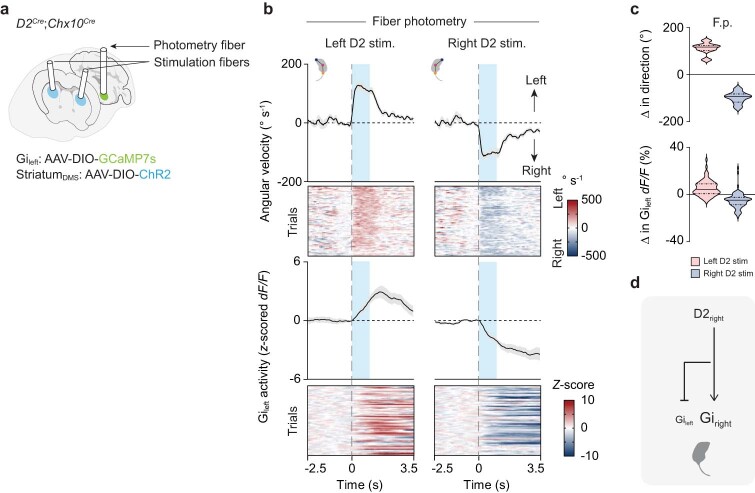
Stereotypic modulation of *Chx10* Gi activity via unilateral activation of D2 SPNs. **a**, Schematic of *Chx10* Gi_left_ GCaMP7s recording with optogenetic stimulation of left or right D2 SPNs. **b**, Stimulation of D2 SPNs caused ipsiversive turns, with an increase in *Chx10* Gi activity ipsilateral to the stimulation and a decrease in *Chx10* Gi activity contralateral to the stimulation. *n* = 7 mice from two independent experiments; 10 left and right D2 stimulation trials for each mouse. Error bands represent the standard error of the mean. **c**, *Top*, Change in direction associated with 1 s ChR2 stimulation of left or right D2 SPNs. *Bottom*, Change in *Chx10* Gi_left_ *ΔF/F* (d*F*/*F*) activity associated with 1-s ChR2 stimulation of left or right D2 SPNs. The magnitude of the change in *ΔF/F* was similar for D2_right_ versus D2_left_ stimulation trials. *P* = 0.81; two-tailed paired t-test; *n* = 7 mice from two independent experiments. Violin plots give the median, the 25th and 75th percentiles, and the range. See Supplementary Table 1 for full statistical analysis. **d**, Model for locomotor asymmetries caused by stimulation of D2 SPNs. Optogenetic stimulation of D2 SPNs has a contralateral inhibitory effect on *Chx10* Gi neurons, as well as an ipsilateral excitatory effect.

Hemispheric imbalances in the activity of dopamine receptor 1 (D1) or dopamine receptor 2 (D2) striatal projection neurons (SPNs) induce locomotor gait asymmetries^2,39,40^. We established a paradigm for studying whether such gait asymmetries generated by the striatum are encoded by *Chx10* Gi activity: GCaMP Ca^2+^ recording was used to assay *Chx10* Gi_left_ neuronal activity in response to optogenetic (channelrhodopsin-2 (ChR2)) stimulation of D1 or D2 SPNs on the left or right side (Fig. 2a and Extended Data Figs. 1 and 2). Here, we obtained selective expression of ChR2 in either D1 or D2 SPNs while recording *Chx10* Gi calcium activity by leveraging the observation that the *D1*^*Cre*^ and *D2*^*Cre*^ alleles exhibit expression in the striatum and not Gi, whereas the *Chx10*^*Cre*^ allele exhibits expression in the Gi and not striatum (Extended Data Fig. 1a–c). This enabled distinct targeting of these populations with viral vectors in *D1*^*Cre*^;*Chx10*^*Cre*^ or *D2*^*Cre*^;*Chx10*^*Cre*^ dual-allelic mice (Extended Data Fig. 1d,e).

In accordance with previous experiments^2,39^, stimulation of right D1 SPNs in the dorsomedial striatum (DMS) caused left turns (contraversive turns; Fig. 2b–e and Supplementary Video 3). These left turns were accompanied by a marked increase in *Chx10* Gi_left_ Δ*F/F* activity at the population level and at the level of single neurons (Fig. 2b–e and Supplementary Video 3). Stimulation of left D1 SPNs initiated right turns associated with a decrease in *Chx10* Gi_left_ Δ*F/F* activity (Fig. 2b–e and Supplementary Video 3). Conversely, stimulation of left D2 SPNs caused left (ipsiversive) turns accompanied by an increase in *Chx10* Gi_left_ population Ca^2+^ activity, whereas stimulation of right D2 SPNs caused right turns associated with a decrease in *Chx10* Gi_left_ Δ*F/F* activity (Extended Data Fig. 2a–c and Supplementary Video 3). These data show that unilateral stimulation of D1 or D2 SPNs modulates *Chx10* Gi activity bilaterally; stimulation of D1 SPNs caused both an increase in Δ*F/F* activity of *Chx10* Gi neurons on the contralateral side and a decrease in *Chx10* Gi activity on the ipsilateral side. The magnitude of the change in Δ*F/F* activity was greater for contralateral D1 stimulation trials (Fig. 2c–e), suggesting that a crossed pathway is dominant in driving the motor action. Together, these data demonstrate that *Chx10* Gi neurons execute basal ganglia-evoked turns, offering parsimonious models as to how this is coordinated by descending *Chx10* reticulospinal projections (Fig. 2f and Extended Data Fig. 2d).

### Pontine commissural neurons link nigral output to *Chx10* Gi

**Fig. 3:**
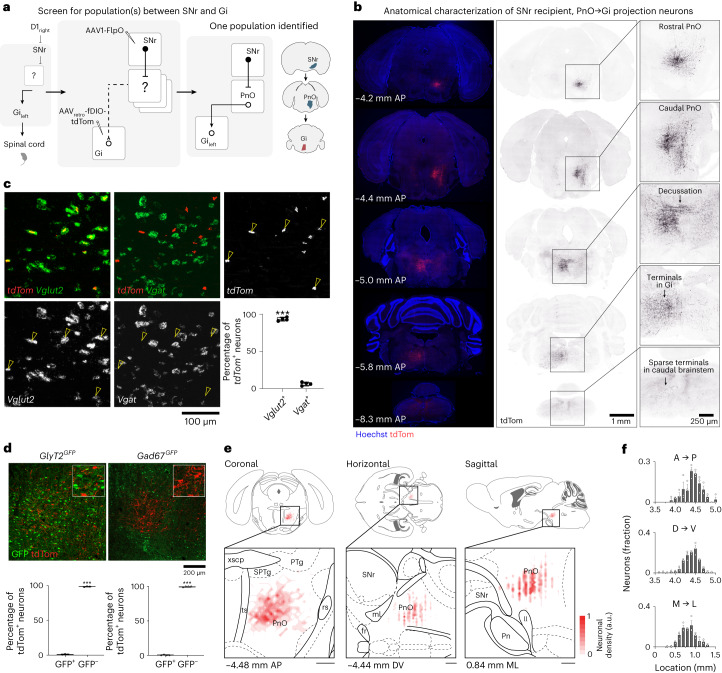
Commissural *Vglut2*^+^ neurons in PnO link SNr to Gi. **a**, Viral screening strategy for identifying link between SNr and Gi. AAV_retro_-fDIO-tdTom was injected in Gi_left_, followed by injection of AAV1-FlpO in SNr_right_. This screen uncovered only one neuronal population which links SNr to Gi located in the PnO (PnO_right_, ipsilateral to SNr and contralateral to Gi). **b**, Anatomy of SNr recipient, PnO → Gi projection neurons. Neurons exhibit caudally projecting axons which decussate at −5.0 mm AP and terminate in the contralateral Gi. Caudal brainstem sections exhibit only sparse axonal projections. Images are representative of *n* = 6 mice from 2 independent experiments. **c**, Triple in situ hybridization for *tdTom*, *Vglut2* and *Vgat* indicates 93.4 ± 1.2% of *tdTom*^+^ (SNr recipient, PnO → Gi projection) neurons co-expressed *Vglut2* whereas 6.6 ± 1.2% of *tdTom*^+^ neurons co-expressed *Vgat*. ***P = 4.2 × 10^−5^; two-tailed paired *t*-test; *n* = 4 mice from 1 experiment. See also Supplementary Table 1. **d**, tdTom labeling of SNr recipient, PnO → Gi projection neurons in *GlyT2*^*GFP*^ or *Gad67*^*GFP*^ mice. SNr recipient, PnO → Gi projection (*tdTom*^+^) neurons are predominantly GFP^−^, indicating they are not glycinergic or GABAergic. *GlyT2*^*GFP*^, ***P = 3.0 × 10^−5^; *Gad67*^*GFP*^, ***P = 4.0 × 10^−7^; two-tailed paired *t*-test; *n* = 3 mice from 1 experiment for *GlyT2*^*GFP*^ and *n* = 4 mice from 2 independent experiments for *Gad67*^*GFP*^. Box-and-whisker plots in **c** and **d** give the median, the 25th and 75th percentiles and the range. **e**, Density plots representing the location of soma in the coronal, horizontal and sagittal planes. Population data are superimposed on plates redrawn from Paxinos and Franklin’s reference atlas^37^. Plates were selected based on the mean AP, DV or ML value of all PnO-Vglut2_contra_ neurons registered. **f**, Quantification of PnO-Vglut2_contra_ neurons in the AP, DV and ML axis relative to bregma, *n* = 3 mice. Error bars in **f** give the s.e.m.

The observation that *Chx10* Gi neurons exhibit stereotyped responses to unilateral activation of D1 or D2 SPNs (Fig. 2 and Extended Data Fig. 2) suggests a discrete pathway linking basal ganglia output, that is, the substantia nigra pars reticulata (SNr), to *Chx10* Gi. However, our previous monosynaptic rabies tracing showed that *Chx10* Gi neurons do not receive direct input from SNr or any other basal ganglia neurons^23^, indicating that the basal ganglia act on *Chx10* Gi neurons via an intermediate pathway link (Fig. 3a).

To identify candidate neuronal population(s) that link the basal ganglia with *Chx10* Gi neurons, we used an intersectional viral screening strategy (Fig. 3a). We targeted SNr as the dominant output nucleus of the basal ganglia^1^. A retrograde virus, AAV_retro_-fDIO-tdTom, was injected in Gi_left_ followed by an anterograde transsynaptic tracer^2,41,42^, AAV1-FlpO, injected in SNr_right_ (Fig. 3a). Here, AAV1 transduces downstream targets of SNr with FlpO, and if these target neurons also project to Gi, they will be labeled with tdTom. This screen uncovered only one cluster of neurons within the anatomical boundaries of the PnO^37^ (PnO_right_, ipsilateral to SNr and contralateral to Gi), which is also known as the rostral pontine reticular nucleus (PRNr) (Fig. 3a,b)^43^. SNr recipient, PnO → Gi projection neurons are commissural, exhibiting descending axons that cross the midline at the level of the pontine reticular nucleus, caudal part (PnC), and which then descend further and arborize in the contralateral Gi (Fig. 3b). Identification of neurons in PnO is consistent with our previous work, which identified PnO as one of ~40 nuclei with monosynaptic input to *Chx10* Gi^23^.

PnO is composed of multiple excitatory and inhibitory projection subtypes (Fig. 3c,d). Using triple in situ hybridization for *tdTom*, vesicular glutamate transporter (*Vglut2*) and vesicular inhibitory amino acid transporter (*Vgat*), we found that 93.4 ± 1.2% of SNr recipient, PnO → Gi projection neurons were *Vglut2* positive and *Vgat* negative (Fig. 3c), indicating a predominantly glutamatergic identity. Indeed, a vast majority of SNr recipient, PnO → Gi projection neurons lacked GFP expression in *GlyT2*^*GFP*^ or *Gad67*^*GFP*^ mice (Fig. 3d). We henceforth refer to this subpopulation of PnO projection neurons as PnO-Vglut2_contra_. Together, these data show that inhibitory basal ganglia output neurons in SNr connect to excitatory *Chx10* Gi neurons^23,24^ via a crossed excitatory glutamatergic hub located in the brainstem PnO.

**Fig. 4:**
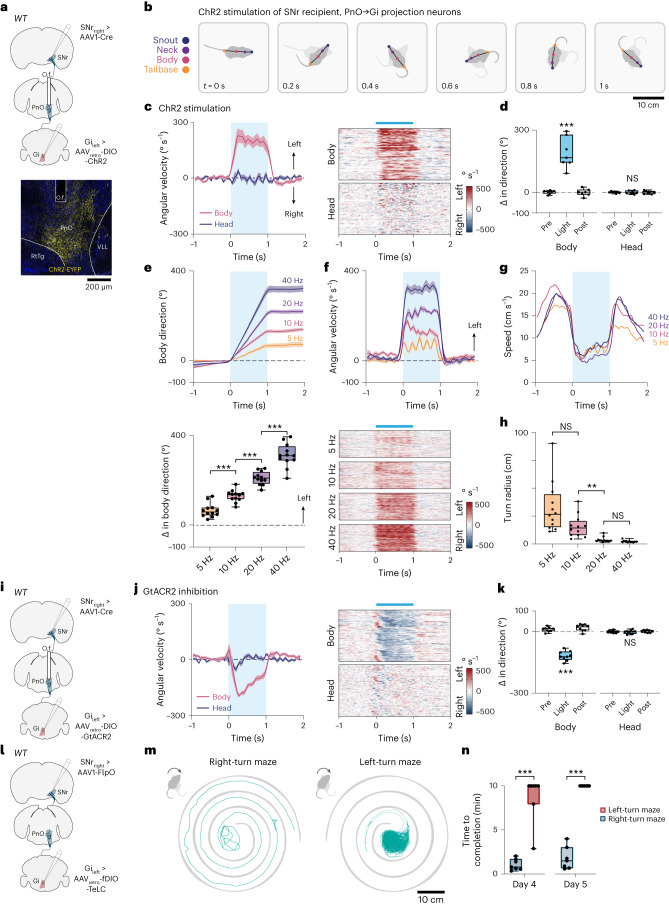
Selective manipulation of PnO-Vglut2_contra_ neurons modulates turning bidirectionally. **a**, Strategy for ChR2 stimulation of PnO-Vglut2_contra_ neurons. Example of ChR2-EYFP expression and optic fiber (O.f.) implantation in the right PnO. Image is representative of *n* = 19 mice from 4 independent experiments. **b**, ChR2 stimulation of PnO-Vglut2_contra_ neurons caused contralateral rotation of the body with little effect on head or trunk (axial) posture. **c**, Angular velocity of the body and head in response to 1-s ChR2 stimulation of PnO-Vglut2_contra_ neurons. **d**, Quantification. Body, ***P = 9.0 × 10^−4^; head, *P* = 0.96; one-way ANOVA with Tukey’s multiple comparison test; *n* = 7 mice from 2 independent experiments. **e**, Change in body direction associated with 1-s ChR2 stimulation. 5 Hz versus 10 Hz, ***P = 6.1 × 10^−5^; 10 Hz versus 20 Hz, ***P = 7.6 × 10^−6^; 20 Hz versus 40 Hz, ***P = 3.1 × 10^−5^; one-way repeated measures ANOVA with Tukey’s multiple comparison test; *n* = 12 mice from 2 independent experiments. **f**, Top, average body angular velocity associated with 1-s ChR2 stimulation. Bottom, trial-by-trial analysis of angular velocity. **g**, ChR2 stimulation was accompanied by a reduction in locomotor speed independent of the frequency of stimulation (see ref. ^23^). *n* = 12 mice from 2 independent experiments. **h**, Average turning radius during 1-s ChR2 stimulation at different frequencies. 5 Hz versus 10 Hz, no significance; 10 Hz versus 20 Hz, **P = 0.0089; 20 Hz versus 40 Hz, no significance; one-way repeated measures ANOVA with Tukey’s multiple comparison test; *n* = 12 mice from 2 independent experiments. **i**, Strategy for GtACR2 inhibition of PnO-Vglut2_contra_ neurons. **j**, Angular velocity of the body and head in response to GtACR2 inhibition of PnO-Vglut2_contra_ neurons. **k**, Quantification. Body, ***P = 4.4 × 10^−7^; head, *P* = 0.94; one-way ANOVA with Tukey’s multiple comparison test; *n* = 9 mice from 2 independent experiments. Error bands in **c**, **e**, **f** and **j** represent the s.e.m. **l**, Strategy for inhibition of PnO-Vglut2_contra_ neurons with TeLC. **m**, TeLC caused ipsilateral turning, and impaired exploration of a contralateral (left-turn) maze. **n**, Quantification. Day 4, ***P = 6.0 × 10^−4^; day 5, ***P = 2.8 × 10^−6^; two-tailed paired *t*-test; *n* = 7 mice from 1 experiment. Box-and-whisker plots in **d**, **e**, **h**, **k** and **n** give the median, the 25th and 75th percentiles and the range. NS, not significant; RtTg, reticulotegmental nucleus of the pons; VLL, ventral nucleus of the lateral lemniscus.

**Extended Data Fig. 3:**
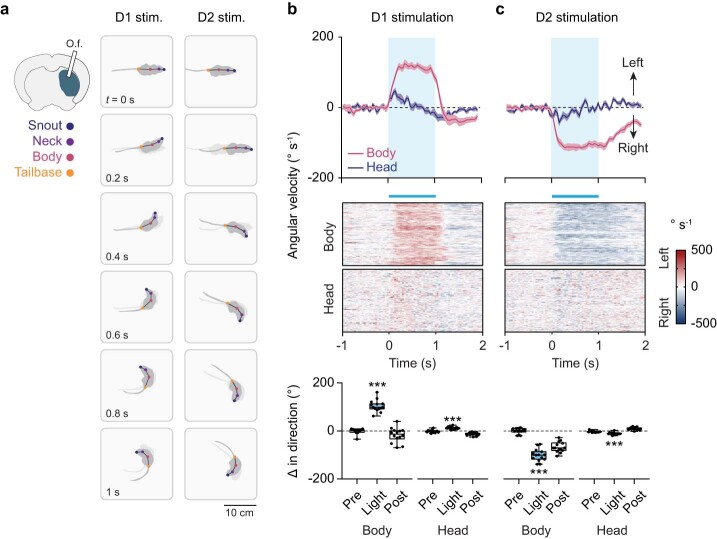
Kinematic analysis for D1 and D2 SPN stimulation experiments. **a**, ChR2 stimulation of D1 or D2 SPNs caused rotation accompanied by contraction of the axial (head and trunk) musculature. For D1 SPNs ChR2-induced rotation was contraversive, whereas for D2 SPNs the rotation was ipsiversive. Contraction of axial musculature is interpreted as a change in the head angle relative to the upper body (contraction of the splenius capitis), or as a change in the angle of the upper body relative to the lower body (contraction of trunk musculature, including the abdominal obliques). Representative example from *n* = 14 fibers implanted in 7 mice from two independent experiments. **b**, Stimulation of D1 SPNs causes changes in both body and head orientation. Body, ***P = 7.7 × 10^−10^; head, ***P = 4.5 × 10^−4^; one-way ANOVA with Tukey’s multiple comparison test; *n* = 14 fibers implanted in 7 mice (where *n* is the average of 10 trials for each fiber) from two independent experiments. **c**, Stimulation of D2 SPNs causes changes in both body and head orientation. Body, ***P = 5.4 × 10^−9^; head, ***P = 6.4 × 10^−4^; one-way ANOVA with Tukey’s multiple comparison test; *n* = 14 fibers implanted in 7 mice (where *n* is the average of 10 trials for each fiber) from two independent experiments. Data in **a-c** represent extended analysis of the data presented in Fig. 2c, Extended Data Fig. 2b. Error bands in the time series plots in **b** and **c** (*top*) represent the standard error of the mean. Box-and-whisker plots in **b** and **c** give the median, the 25th and 75th percentiles, and the range.

**Extended Data Fig. 4:**
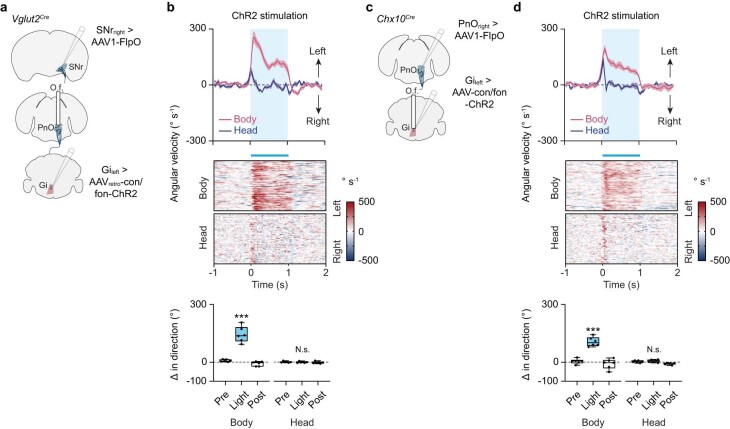
Projection and cell type-specific ChR2 interrogation of the PnO → Gi pathway. **a**, Strategy for stimulating Vglut2^+^ neurons in PnO which receive input from SNr_ipsi_ and project to Gi_contra_. In *Vglut2*^*Cre*^ mice, AAV_retro_-con/fon-ChR2 was injected in Gi_left_ followed by AAV1-FlpO in SNr_right_. An optic fiber (O.f.) was then implanted over PnO. **b**, Optogenetic stimulation of PnO-Vglut2_contra_ neurons using the strategy in (**a**) evoked contraversive turning. Body, ***P = 5.7 × 10^−4^; head, *P* = 0.90; one-way ANOVA with Tukey’s multiple comparison test; *n* = 6 mice (where *n* is the average of 10 trials for each mouse) from one experiment. **c**, Strategy for stimulating Chx10^+^ neurons in Gi which receive input from the PnO_contra_. In *Chx10*^*Cre*^ mice, AAV1-FlpO was injected in PnO_right_ followed by AAV-con/fon-ChR2 in Gi_left_. An optic fiber was then implanted over Gi. **d**, Optogenetic stimulation of PnO recipient, Chx10^+^ Gi neurons evoked ipsiversive turning. Body, ***P = 1.3 × 10^−4^; head, *P* = 0.80; one-way ANOVA with Tukey’s multiple comparison test; *n* = 6 mice (where *n* is the average of 10 trials for each mouse) from one experiment. Error bands in the time series plots in **b** and **d** (*top*) represent the standard error of the mean. Box-and-whisker plots in **b** and **d** give the median, the 25th and 75th percentiles, and the range.

To assess the possibility at a behavioral level that PnO-Vglut2_contra_ neurons are involved in turning, we performed gain- and loss-of-function optogenetic studies using ChR2 or GtACR2 (Fig. 4a). ChR2 stimulation (40 Hz) of PnO-Vglut2_contra_ neurons produced robust turning toward the contralateral side (Fig. 4b–d and Supplementary Video 4), corresponding to crossed excitatory action on *Chx10* Gi_contra_ neurons. Notably, in contrast to stimulation of D1 or D2 SPNs (Extended Data Fig. 3 and Supplementary Video 3), ChR2-mediated stimulation of PnO-Vglut2_contra_ neurons evoked contralateral turning of the body with limited effect on axial (head or trunk) posture (Fig. 4b–d and Extended Data Fig. 4). Varying the stimulation frequency allowed tight control of turning kinematics, with lower stimulation frequencies (5–20 Hz) inducing smaller changes in angular velocity accompanied by larger turning radii (Fig. 4e–h and Supplementary Video 5). To confirm that this phenotype was linked to a glutamatergic identity, we used an INTRSECT strategy^44^ to express ChR2 in PnO-Vglut2_contra_ neurons based both on input (SNr_ipsi_)/output (Gi_contra_) connectivity and on Vglut2 identity (Extended Data Fig. 4a). Stimulation of PnO-Vglut2_contra_ neurons using this approach recapitulated the contralateral turning phenotype (Extended Data Fig. 4b). Furthermore, using an INTRSECT strategy, we confirmed that *Chx10* Gi neurons act as the postsynaptic target of PnO-Vglut2_contra_ neurons (Extended Data Fig. 4c,d). Finally, GtACR2 photoinhibition of PnO-Vglut2_contra_ neurons caused robust turning toward the ipsilateral side (Fig. 4i–k and Supplementary Video 4). This latter experiment indicates that PnO-Vglut2_contra_ neurons are tonically active at rest, such that inhibitory tone from SNr would modulate the activity of PnO-Vglut2_contra_ neurons bidirectionally: decreased SNr activity would increase the activity of PnO-Vglut2_contra_ neurons, whereas increased SNr activity would decrease the activity of PnO-Vglut2_contra_ neurons. To evaluate the necessity of the PnO → Gi pathway for turning, we chronically silenced PnO-Vglut2_contra_ neurons using viral-mediated expression of tetanus-toxin light chain (TeLC; Fig. 4l). TeLC expression caused strong ipsilateral (right) turning within 4 d of viral delivery (Fig. 4l–n). Mice were then tested in spiral-shaped mazes (Fig. 4m), which the mice were allowed to explore to completion or until 10 min had elapsed. Unaffected mice rapidly completed both left- and right-turn mazes^23^. In contrast, mice with chronic silencing of PnO-Vglut2_contra_ neurons readily completed the right-turn (ipsilateral) maze but could not complete the left-turn (contralateral) maze (Fig. 4l–n). These experiments show that mice cannot compensate for a loss of PnO-Vglut2_contra_ function; PnO-Vglut2_contra_ neurons are requisite for natural exploratory behavior.

### Nigral-mediated turning gaits act predominantly via PnO

**Fig. 5:**
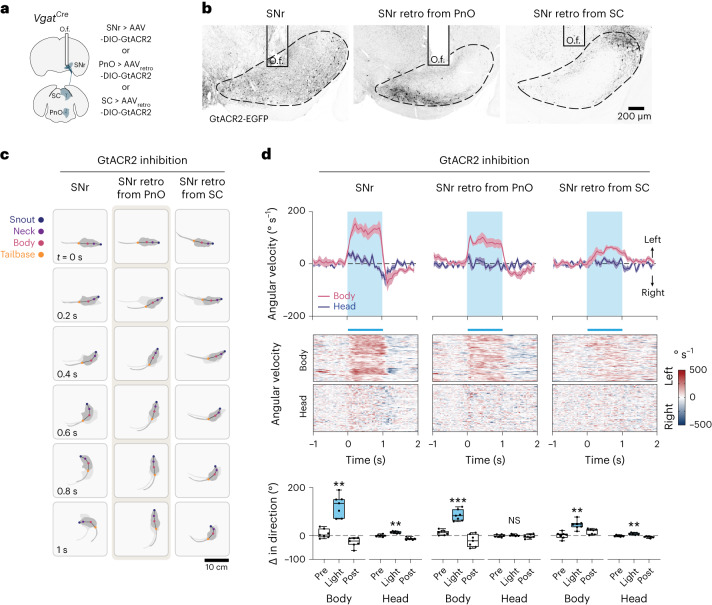
Basal ganglia turning gait asymmetries act predominantly via PnO. **a**, Strategies for GtACR2 transduction of a bulk Vgat^+^ SNr population, or specific populations of Vgat^+^ SNr → PnO or SNr → SC projection neurons. **b**, Examples of GtACR2 transduction and fiber placement using strategies in **a**. Vgat^+^ neurons labeled via retrograde transduction from PnO exhibited localization within a caudomedial domain, whereas Vgat^+^ neurons labeled via retrograde transduction from SC exhibited localization within a rostrolateral domain. Images are representative of *n* = 7 mice from 2 independent experiments for each condition. **c**, Optogenetic inhibition of SNr caused limb-based changes in body orientation, as well as contraction of the trunk musculature. Selective optogenetic inhibition of SNr → PnO projection neurons evoked a strong limb-based contralateral rotation of the body without a prominent effect on axial (head or trunk) posture. Selective optogenetic inhibition of SNr → SC projection neurons caused weak limb-based changes in body orientation, as well as contraction of the trunk musculature. **d**, Left, inhibition of a bulk Vgat^+^ SNr population. Body, **P = 0.0013; head, **P = 0.002; one-way ANOVA with Tukey’s multiple comparison test. Center, selective inhibition of SNr → PnO projection neurons. Body, ***P = 1.8 × 10^−4^; head, *P* = 0.70; one-way ANOVA with Tukey’s multiple comparison test. Right, selective inhibition of SNr → SC projection neurons. Body, **P = 0.0013; head, **P = 0.0056; one-way ANOVA with Tukey’s multiple comparison test. *n* = 7 mice from 2 independent experiments for each condition, where *n* is the average of 10 trials for each mouse. Error bands in the time series plots (top) represent the s.e.m. Box-and-whisker plots give the median, the 25th and 75th percentiles and the range.

We undertook a series of optogenetic studies to further understand the SNr → PnO projection. Previous work has demonstrated that broad inhibition of SNr neurons induces contralateral turning^45,46^. To confirm and benchmark this phenotype, we broadly transduced SNr with AAV-DIO-GtACR2 (*Vgat*^*Cre*^ > DIO-GtACR2; Fig. 5a,b). Broad GtACR2 inhibition of SNr neurons produced robust contralateral rotation which consisted of changes in body orientation mediated by the limbs as well as contraction of the axial head and trunk musculature (Fig. 5c,d). Compared with broad SNr transduction, retrograde transduction of SNr by injection of AAV_retro_-DIO-GtACR2 in PnO labeled only a subpopulation of SNr neurons localized within a caudomedial domain (Fig. 5b). GtACR2 inhibition of this restricted SNr → PnO population evoked contralateral turning, accounting for approximately 70% (68.0 ± 7.0%, *n* = 7 mice) of the limb-based turning phenotype obtained by broad SNr inhibition (Fig. 5d). Notably, whereas broad SNr inhibition caused limb-based changes in body orientation as well as contraction of the head and trunk musculature, inhibition of the restricted SNr → PnO population evoked only a limb-based contralateral rotation of the body without a prominent effect on axial (head or trunk) posture (Fig. 5c,d and Supplementary Video 6).

Broad activation of SNr likely recruits axial motor networks via projections to the superior colliculus (SC)^47,48^. To investigate this possibility, we retrogradely transduced SNr by injection of AAV_retro_-DIO-GtACR2 in SC, labeling a subpopulation of SNr neurons localized within a rostrolateral domain (Fig. 5b). GtACR2 inhibition of this restricted SNr → SC population evoked contralateral turning, and recapitulated changes in head and trunk posture that were associated with broad SNr inhibition (Fig. 5c,d). Notably, the limb-based turning phenotype associated with inhibition of SNr → SC neurons accounted for only 35.8 ± 5.6% (*n* = 7 mice) of that associated with broad SNr inhibition, and was substantially reduced compared with specific SNr → PnO inhibition (Fig. 5d). These data indicate that limb-based changes in body orientation can be largely dissociated from turning of the head at the level of brainstem motor circuits; the SNr → PnO pathway controls limb-based body orientation, whereas the SNr → SC pathway primarily controls head orientation. The outputs of these two pathways cross the midline and re-converge at the level of reticulospinal populations in the contralateral Gi, where *Chx10* subpopulation(s) control limb-based body orientation and/or head orientation, respectively (Extended Data Fig. 4c,d)^23,28^.

**Extended Data Fig. 5:**
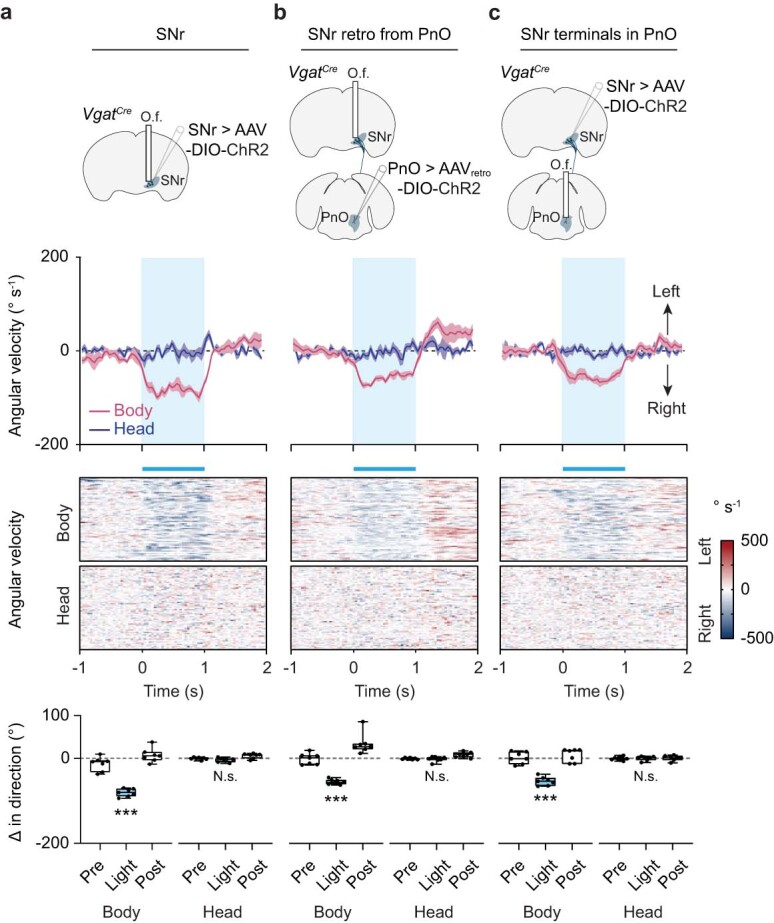
ChR2 interrogation of the Vgat^+^ SNr → PnO projection. **a**, ChR2 stimulation of Vgat^+^ SNr neurons caused ipsiversive turning. Body, ***P = 1.8 × 10^−4^; head, *P* = 0.43; one-way ANOVA with Tukey’s multiple comparison test; *n* = 7 mice (where *n* is the average of 10 trials for each mouse) from two independent experiments. **b**, The ipsiversive turning phenotype using the strategy in (**a**) was partly recapitulated by stimulating Vgat^+^ neurons in SNr that were retrogradely targeted from PnO, Body, ***P = 5.4 × 10^−5^; head, *P* = 0.95; one-way ANOVA with Tukey’s multiple comparison test; *n* = 7 mice (where *n* is the average of 10 trials for each mouse) from two independent experiments. **c**, The ipsiversive turning phenotype using the strategy in (**a**) was partly recapitulated by stimulating Vgat^+^ SNr terminals in PnO. Body, ***P = 8.3 × 10^−5^, head, *P* = 0.98; one-way ANOVA with Tukey’s multiple comparison test; *n* = 7 mice (where *n* is the average of 10 trials for each mouse) from two independent experiments. Error bands in the time series plots (*top*) represent the standard error of the mean. Box-and-whisker plots give the median, the 25th and 75th percentiles, and the range.

Mirroring the effect of GtACR2 inhibition, broad optogenetic activation of inhibitory SNr neurons produced ipsilateral turning (Extended Data Fig. 5a). ChR2 stimulation of inhibitory SNr neurons retrogradely transduced from PnO accounted for approximately 70% (68.5 ± 2.9%) of the limb-based ipsilateral turning phenotype obtained by directly targeting SNr (Extended Data Fig. 5b). Notably, this effect was likely mediated through SNr → PnO projections and not via potential collaterals since stimulation of inhibitory SNr terminals in PnO also evoked ipsilateral turning (Extended Data Fig. 5c), accounting for 66.0 ± 4.7% of the phenotype associated with broad SNr stimulation.

Together, these data show that basal ganglia-evoked turning gait asymmetries act predominantly through neurons located in PnO. Moreover, the data show that the SNr → PnO basal ganglia output channel is specific for limb-based turning mechanisms which act to promote hindlimb extensor tone, rather than those mechanisms that control axial bending (see ref. ^23^).

### Reversal of dopamine depletion-induced gait asymmetries

Gait deficits are a defining characteristic of Parkinson’s disease^32,33^. In advanced Parkinson’s disease, turning deficits represent a hallmark symptom, characterized by increased turning duration, an increased number of small steps required to complete a turn and impaired rotational coordination^30,31,35,36^. The motor substrate for turning deficits in Parkinson’s disease is unknown. However, unilateral hypoactivity in the PnO → *Chx10* Gi pathway may help to explain the limb-based rotational phenotypes observed in hemi-parkinsonism^49^. We therefore set out to investigate this in a hemi-parkinsonian model in mice. For this, we lesioned the nigrostrial pathway via unilateral administration of the catecholaminergic neurotoxin 6-hydroxydopamine (6-OHDA), which leads to increased turning toward the lesioned side and an exacerbated turning deficit toward the contralateral side^50–53^. In this model we attempted to reverse 6-OHDA-induced gait asymmetries by modulation of the downstream PnO → *Chx10* Gi pathway.

**Fig. 6:**
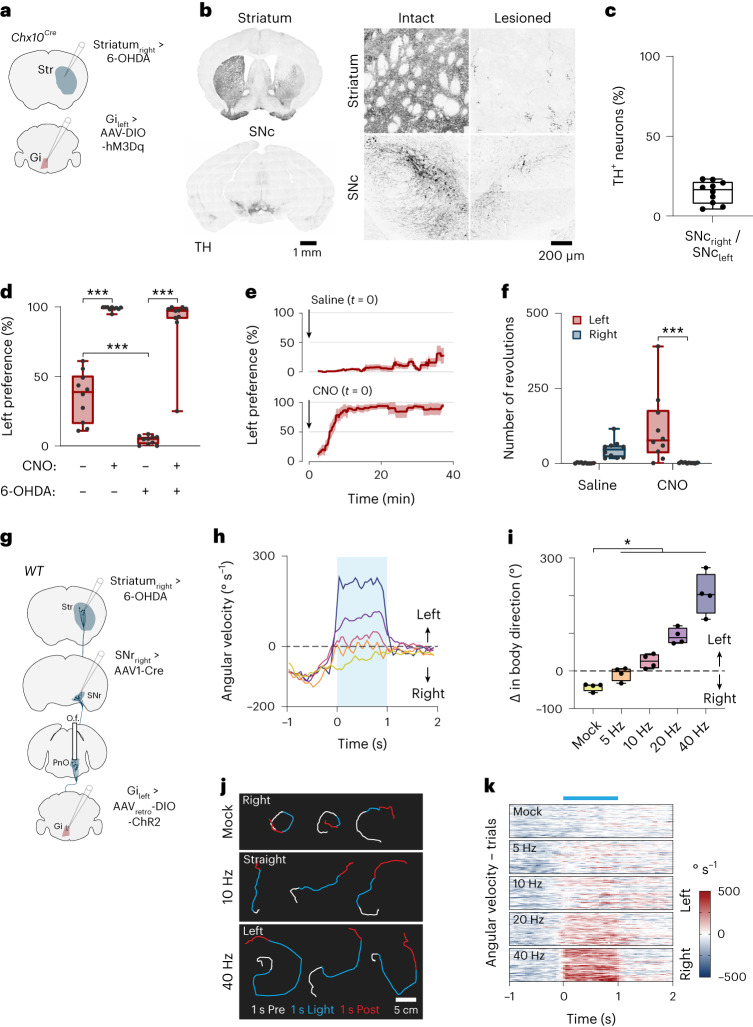
Restoration of contraversive turning gaits in mice with acute unilateral striatal dopamine depletion. **a**, Strategy for restoring contraversive turning in mice with unilateral striatal dopamine depletion. **b**, Unilateral 6-OHDA-lesioned mice exhibited loss of dopaminergic (TH-positive, TH^+^) terminals in the ipsilateral striatum, and loss of TH^+^ neurons of the ipsilateral SNc. **c**, Quantification of 6-OHDA lesion efficacy. *n* = 10 mice from 2 independent experiments. **d**, Analysis of left versus right movement preference in the open field. −CNO/−6-OHDA versus +CNO/−6-OHDA, ***P = 8.3 × 10^−11^; −CNO/−6-OHDA versus −CNO/+6-OHDA, ***P = 1.7 × 10^−4^; −CNO/+6-OHDA versus +CNO/+6-OHDA, ***P < 1.0 × 10^−15^; two-way ANOVA with Tukey’s multiple comparison test; *n* = 10 mice from 2 independent experiments. Pre-lesion data (−6-OHDA) are also presented in Extended Data Fig. 6d. **e**, Mice with acute unilateral striatal dopamine depletion exhibited predominantly ipsiversive (right) movement preference. CNO administration at *t* = 0 reversed ipsiversive rotational biases in a time-dependent manner. Error bands represent the s.e.m. **f**, CNO left versus CNO right, ***P = 9.0 × 10^−4^; two-way ANOVA with Tukey’s multiple comparison test; *n* = 10 mice from 2 independent experiments. **g**, Strategy for testing the efficacy of PnO-Vglut2_contra_ neurons in restoring contraversive turning in mice with acute unilateral striatal dopamine depletion. **h**, Optogenetic stimulation of PnO-Vglut2_contra_ neurons restored straight locomotion at 5 Hz and 10 Hz and contralateral turning at 20 Hz and 40 Hz. **i**, Mock versus 5 Hz, *P = 0.02; mock versus 10 Hz, *P = 0.018; mock versus 20 Hz, *P = 0.012; mock versus 40 Hz, *P = 0.018; one-way repeated measures ANOVA with Tukey’s multiple comparison test; *n* = 4 mice (where *n* is the average of 10 trials for each mouse) from 1 experiment. **j**, Example trajectories associated with mock or 1-s ChR2 stimulation. **k**, Trial-by-trial analysis of angular velocity. *n* = 4 mice (10 trials per mouse) from 1 experiment. Box-and-whisker plots in **c**, **d**, **f** and **i** give the median, the 25th and 75th percentiles and the range.

**Extended Data Fig. 6:**
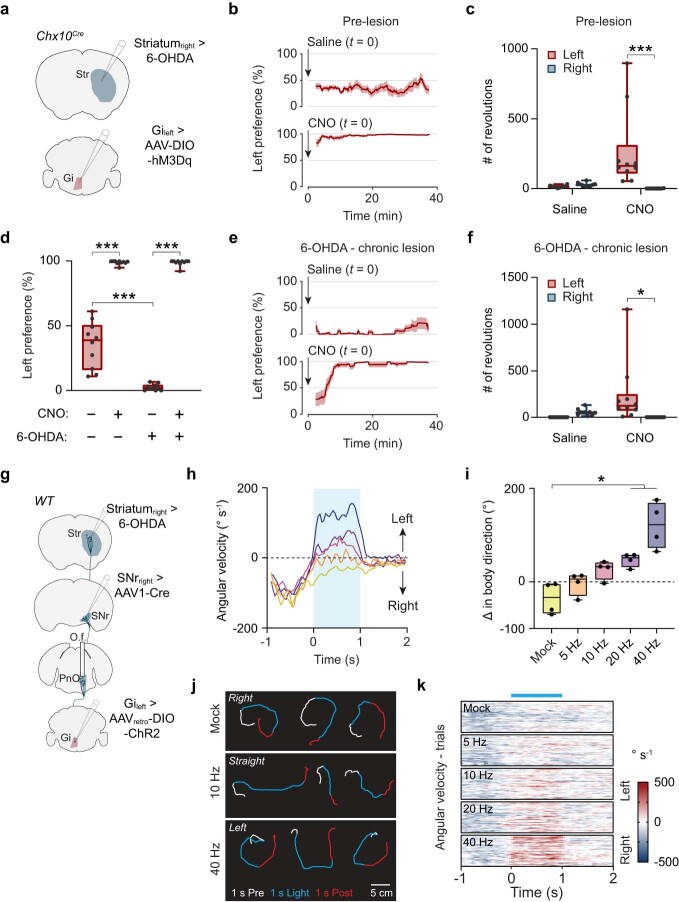
Restoration of contraversive turning gaits in mice with chronic unilateral striatal dopamine depletion. **a**, Strategy for restoring contraversive turning in mice with unilateral striatal dopamine depletion. (**b-c**) Pre-lesion testing of *Chx10*^*Cre*^ mice with Cre-dependent expression of hM3Dq-DREADDs in Gi_left_. **b**, Analysis of left vs. right movement preference in the open field **c**, CNO left vs. CNO right, ***P = 9.0 × 10^−4^; two-way ANOVA with Tukey’s multiple comparison test; *n* = 10 mice from two independent experiments. **d**, Mice with chronic unilateral striatal dopamine depletion exhibited a loss of contraversive (left) turning gaits (–CNO/+6-OHDA). CNO administration (+CNO/+6-OHDA) reversed ipsiversive (right) rotational biases and restored contraversive (left) turning gaits. –CNO/–6-OHDA vs. +CNO/–6-OHDA, ***P < 1.0 × 10^−15^; –CNO/–6-OHDA vs. –CNO/+6-OHDA, ***P = 8.6 × 10^−9^; –CNO/+6-OHDA vs. +CNO/+6-OHDA, ***P < 1.0 × 10^−15^; two-way ANOVA with Tukey’s multiple comparison test; *n* = 10 mice from two independent experiments. Pre-lesion data (–6-OHDA) is also presented in Fig. 6d. **e**, Mice with chronic unilateral striatal dopamine depletion exhibited predominantly ipsiversive (right) movement preference. CNO administration at *t* = 0 reversed ipsiversive rotational biases in a time-dependent manner. Error bands in **b** and **e** represent the standard error of the mean. **f**, CNO left vs. CNO right, *P = 0.017; two-way ANOVA with Tukey’s multiple comparison test; *n* = 10 mice from two independent experiments. **g**, Strategy for testing the efficacy PnO-Vglut2_contra_ neurons in restoring contraversive turning in mice with chronic unilateral striatal dopamine depletion. **h**, Optogenetic stimulation of PnO-Vglut2_contra_ neurons restored straight locomotion at 5 Hz and 10 Hz and contraversive turning at 20 Hz and 40 Hz. **i**, Mock vs. 20 Hz, *P = 0.021; Mock vs. 40 Hz, *P = 0.029; one-way repeated measures ANOVA with Tukey’s multiple comparison test; *n* = 4 mice from one experiment. **j**, Example trajectories associated with mock or 1-s ChR2 stimulation at 10 Hz or 40 Hz. **k**, Trial-by-trial analysis of angular velocity. *n* = 4 mice from one experiment. Box-and-whisker plots in **c**, **d**, **f**, and **i** give the median, the 25th and 75th percentiles, and the range.

We first addressed the role of *Chx10* Gi neurons. To stimulate *Chx10* Gi_left_ neurons, *Chx10*^*Cre*^ mice were injected unilaterally with excitatory DREADDS (DIO-hM3Dq) (Fig. 6a and Extended Data Fig. 6a). Under baseline (saline) conditions, mice showed close to symmetric turning. After activation of *Chx10* Gi_left_ neurons with clozapine *N*-oxide (CNO; 1 mg kg^−1^), mice exhibited turning towards the side of stimulation (the left side; see ref. ^23^) (Extended Data Fig. 6b,c). The same mice were then injected with 6-OHDA in the right DMS for acute (4–6 d after injection) or chronic (15–19 d after injection) models of hemi-parkinsonism. Injection of 6-OHDA led to loss of dopaminergic (tyrosine hydroxylase (TH)-positive, TH^+^) terminals in the striatum on the side of injection, and reduction of dopaminergic cell bodies in the ipsilateral substantia nigra pars compacta (SNc) (Fig. 6b,c). Unilateral 6-OHDA-lesioned mice exhibited a dominantly ipsiversive (right) turning preference in both the acute and chronic lesioned states (Fig. 6d–f, Extended Data Fig. 6d–f and Supplementary Video 7). Activation of *Chx10* Gi_left_ neurons with CNO (1 mg kg^−1^) rapidly reversed the ipsiversive turning, restoring contraversive turning gaits (Fig. 6d–f and Extended Data Fig. 6d–f). The reversal developed slowly, with an early phase between 5 min and 10 min after CNO administration where mice exhibited close to symmetric turning, which then developed into predominantly contraversive turning (Fig. 6e, Extended Data Fig. 6e and Supplementary Video 7). These experiments show that turning gait deficits induced by unilateral striatal dopamine depletion can be reversed by activation of *Chx10* Gi neurons.

We next investigated the contribution of PnO-Vglut2_contra_ neurons (Fig. 6g and Extended Data Fig. 6g). Graded ChR2 stimulation (5–40 Hz, pulse duration 15 ms, fixed amplitude) of PnO-Vglut2_contra_ neurons modulates the magnitude of turning in a frequency-dependent manner (Fig. 4e–h). A similar graded stimulation of PnO-Vglut2_contra_ neurons on the lesioned side in the acute or chronic phase of hemi-parkinsonism reversed ipsiversive turning biases, restoring straight locomotion at low frequencies (5 Hz or 10 Hz in the acute and chronic phases) and contraversive turning gaits at high frequencies (20 Hz, 40 Hz) (Fig. 6h–k, Extended Data Fig. 6h–k and Supplementary Video 8). These data show that graded stimulation of PnO-Vglut2_contra_ neurons is sufficient to normalize gait asymmetries and to restore contralateral turning in hemi-parkinsonian mice.

## Discussion

Our study reveals the functional organization of circuits that control left–right turning gait asymmetries. We show how the basal ganglia work in tandem with brainstem circuits to recruit spinal motor networks, providing a detailed account as to how the basal ganglia execute a complex movement. Recent insight that gait asymmetries are definitively controlled via a dedicated population of *Chx10* Gi neurons that project directly to the spinal cord^23,28^ was key to establishing this motor pathway. Our results thus demonstrate how well-known basal ganglia-mediated turning gait asymmetries are produced, opening the possibility of addressing this question in the framework of brain-wide networks.

**Fig. 7:**
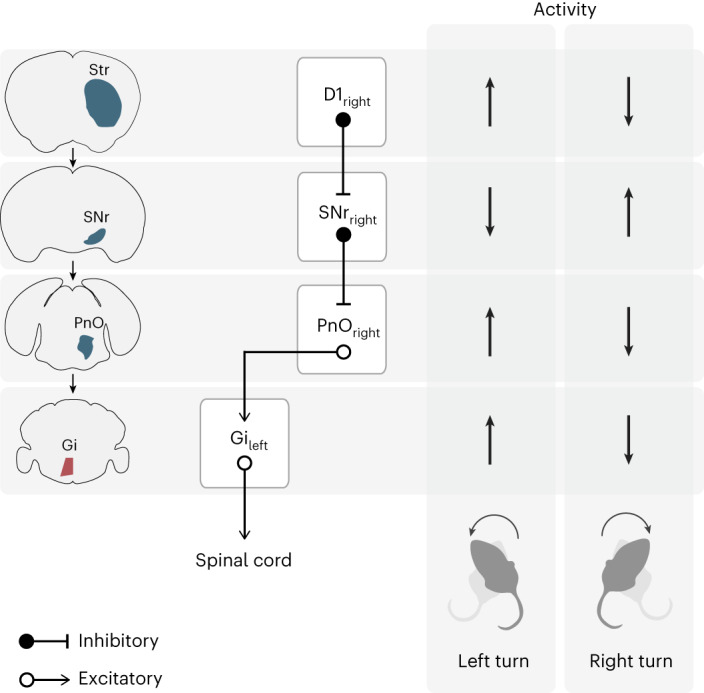
Summary of findings. Activity of D1 SPNs causes contraversive turning gait asymmetries via nigral disinhibition of the PnO → *Chx10* Gi excitatory brainstem–spinal cord pathway^23,39,45^. Loss of D1 SPN activity causes ipsiversive turning via nigral inhibition of the PnO → *Chx10* Gi excitatory brainstem pathway^23,40,55^.

A classic model of basal ganglia control over motor actions holds that opponent pathways, the striatal direct and indirect pathways, enable bidirectional control over downstream motor programs^19,39,54^. In the context of turning gait asymmetries, stimulation of direct pathway SPNs promotes contraversive turning and stimulation of indirect pathway SPNs promotes ipsiversive turning^39^. Furthermore, at the level of SNr, a dominant basal ganglia output, unilateral stimulation of inhibitory neurons promotes ipsiversive turning^55^ and unilateral silencing promotes contraversive turning^45^ (as we confirm here). The simplest model would posit that SNr bidirectionally modulates a specific population of downstream neurons around a setpoint of tonic activity. Indeed, we found that PnO-Vglut2_contra_ neurons can be modulated bidirectionally—through decreased or increased inhibitory input via a specific SNr → PnO channel—initiating either contraversive or ipsiversive turning, respectively. Therefore, decreased unilateral SNr activity caused by unilateral D1 activation would act to disinhibit PnO-Vglut2_contra_ neurons, allowing activation of contralateral *Chx10* Gi to initiate contraversive turns. In contrast, increased SNr activity by unilateral D2 activation would act to inhibit PnO-Vglut2_contra_ neurons, leading to ipsiversive turns (Fig. 7)^23,24,28^.

We observed bidirectional modulation of *Chx10* Gi population activity during spontaneous turns, with increased or decreased activity when the animals turn to the ipsilateral or contralateral side, respectively. Notably, we also observed a bilateral decrease in *Chx10* Gi activity during straight events. While we did not relate population activity to stop events^24^, importantly, changes in population activity were not correlated with other types of active nonlocomotor behaviors such as grooming as previously described from single neuron recordings^56^. The difficulties we encountered in performing reliable single neuron recording in Gi prevented us from addressing this issue further. However, most of the (few) cells we recorded using endoscopic imaging recapitulated the activity patterns observed in population recordings using fiber photometry. *Chx10* Gi neurons themselves enable bidirectional modulation of spinal locomotor network activity^24^ (Fig. 7). Together, these data imply that the mode of operation along the SNr → PnO-Vglut2_contra_ → *Chx10* Gi axis is resting tonic activity that can be decreased or increased to generate turns. Candidate mechanisms for resting tonic activity of PnO-Vglut2_contra_ or *Chx10* Gi neurons include those mechanisms demonstrated for SNr, where tonic activity depends on resting inward conductances^1,57–60^.

We identified PnO-Vglut2_contra_ neurons as a critical link interfacing between basal ganglia output and *Chx10* Gi reticulospinal neurons. Anatomical projection to the PnO from a restricted area of SNr has recently been described^1^. However, the functional role of SNr-PnO projection neurons has not been determined. Various motor functions, including postural adjustment and motor immobility^61^, have been suggested to be regulated by neurons in PnO. These and our data indicate that the greater PnO represents a heterogeneous nucleus composed of multiple excitatory and inhibitory projection neuron subtypes. Uniquely, our study identifies a neuronal pathway from specific SNr neurons that targets a select population of projection neurons within PnO, excitatory neurons which project to the contralateral Gi. This PnO projection subtype exhibits exquisite specificity for limb control, identifying a circuit mechanism for limb-based (body) turning which is recruited by basal ganglia activity^49^. The limb-based turning contrasts with the dominant head-based turning evoked by the SNr → SC pathway. Together, our data indicate that limb-based turning—which is the major factor determining locomotor direction—and head turning may be mediated by separate anatomical and functional pathways at the level of SNr and brainstem motor circuits. These data accord with previous work implicating the SC in the control of head and trunk posture^23,47,62,63^. The SNr → PnO and SNr → SC pathways project ipsilaterally, while the outputs of PnO and SC cross the midline and re-converge in the contralateral Gi^23,63^. Additionally, however, animals (and humans) also require coordination of head and trunk orientation with limb-based turns, and appropriate scaling of these respective signals is likely necessary for gross coordination of movements. One possibility is that SC → Gi projections modulate limb-based turning signals via collaterals at the level of the PnO or Gi.

Finally, we demonstrate that turning deficits caused by unilateral striatal dopamine depletion can be reversed by graded stimulation of the PnO → *Chx10* Gi pathway. From the first experiments using 6-OHDA to mimic dopamine loss after Parkinson’s disease^50,51^, it is well established that unilateral striatal dopamine depletion causes turning towards the lesioned side^51^. Although there has been no clear understanding as to how central dopamine imbalance leads to asymmetric locomotor activity, there is clear evidence of limb-associated changes which drive the action^49^. Our study provides a putative basal ganglia–spinal cord pathway mechanism for locomotor asymmetries deriving from dopamine imbalance between hemispheres, suggesting that asymmetric gait phenotypes arising from such imbalance could be mediated by a loss of activity in the descending excitatory PnO → *Chx10* Gi brainstem–spinal cord pathway (Fig. 7). Turning deficits are a component of the gait disturbances observed in severe Parkinson’s disease^30,31^. Typically, affected individuals cannot perform smooth limb-based turns, and often compensate with small, incremental steps that eventually culminate in a complete turn. We therefore posit that turning deficits may in part be explained by decreased activity in the PnO → *Chx10* Gi pathway as a consequence of increased nigral inhibition of brainstem motor circuits (Fig. 7). Substantiation of this hypothesis would necessitate future studies to provide further accounts as to how activity of the PnO → *Chx10* Gi pathway changes after acute or chronic depletion of dopamine. Given this information, modulation of the PnO → *Chx10* Gi pathway could potentially serve as a target for deep brain stimulation aimed at alleviating turning disabilities in Parkinson’s disease clinically.

## Methods

### Mice

Animal procedures were performed in accordance with European Union Directive 2010/63/EU, and approved by the Danish Animal Inspectorate (Dyreforsøgstilsynet, permits 2017-15-0201-01172 and 2022-15-0201-01131) as well as the clinical veterinarians at the Department of Experimental Medicine, Faculty of Health and Medical Sciences, University of Copenhagen (plans P19-134, P21-323, P22-502, A20-160 and A23-154). Experiments were performed in adult mice greater than 8 weeks of age, with an effort to include similar numbers of male and female mice. The following strains were used for the experiments herein: *Chx10*^*Cre*^ (ref. ^64^), *D1*^*Cre*^ (Gensat EY217), *D2*^*Cre*^ (Gensat ER44), *R26R*^*LSL-tdTom*^ (ref. ^65^) (Ai14, Jackson Stock no. 007914), *WT* (C57BL6/J, Jackson Stock no. 000664), *GlyT2*^*GFP*^ (ref. ^66^), *GAD67*^*GFP*^ (refs. ^67,68^), *Vglut2*^*Cre*^ (ref. ^69^), *Vgat*^*Cre*^ (ref. ^70^ or ref. ^71^, Jackson Stock no. 028862). Mice were housed in ventilated cages with ad libitum access to food and water. Cages were maintained within a temperature- (23–24 °C) and humidity- (45–50%) controlled environment, with a 12-h light/dark cycle.

### Stereotaxic viral injections

Anesthesia was induced with 4% isofluorane (Link 7 Anesthesia & Evacuation System, Patterson Scientific), and then maintained at 1.5–2% during surgery. Mice were secured to a stereotaxic frame (Model 900, David Kopf Instruments) which was driven by a robotic controller (Neurostar). Mice were kept on a 37 °C heating pad (Rodent Warmer X1, Stoelting) for the duration of the surgery. Viscotears were applied to the eyes to prevent dehydration. The skin on the head was shaved, and an incision was made to expose the skull. Skull references were taken for bregma and lambda, and a small burr hole was drilled in the skull overlying the target brain region for injection. The underlying dura was opened. A pulled glass capillary was filled with mineral oil and secured to a capillary nanoinjector (Neurostar). Viruses were mixed with a small amount of Fast Green for visualization and loaded into the capillary. The capillary was advanced to the target brain region at a rate of 0.1 mm s^−1^, and injections were performed at a rate of 100 nl min^−1^. The capillary was left in place for 5 min following the injection and then withdrawn at a rate of 0.1 mm s^−1^. The skin was closed using a series of individual 6-0 sutures (Ethicon) or wound clips (7 mm, Reflex Autoclip System). Mice were allowed to recover on the heating pad, and post-operative buprenorphine (0.3 mg kg^−1^) was given for pain management.

For Ca^2+^ recording from *Chx10* Gi neurons (Figs. 1 and 2 and Extended Data Figs. 1 and 2), AAV1-hSyn1-DIO-GCaMP6s (for endoscopic imaging; 100845-AAV1, 2.0 × 10^13^ ml^−1^, Addgene) or AAV9-hSyn1-DIO-GCaMP7s (for fiber photometry; v407-9, 5.0 × 10^12^ ml^−1^, Viral Vector Facility, University of Zurich) was injected in Gi_left_ (coordinates: −6.0 mm anteroposterior (AP), −0.8 mm mediolateral (ML), −5.5 mm dorsoventral (DV)). The coordinates for injection in Gi are the same as those used in refs. ^23,24,28^. Ca^2+^ recordings were combined with optogenetic stimulation of direct or indirect pathway SPNs in *D1*^*Cre*^;*Chx10*^*Cre*^ or *D2*^*Cre*^;*Chx10*^*Cre*^ dual-allelic mice (Fig. 2 and Extended Data Figs. 1–3). We targeted the DMS based on previous data indicating that turning is most effectively induced by stimulation of the medial sector^2^ (comparable with ref. ^39^), although turning can also be evoked from the dorsolateral striatum^2,40^. For this, AAVdj-Ef1a-DIO-hChR2(E123T/T159C)-p2A-mCherry-WPRE (GVVC-AAV-33, 9.0 × 10^12^ ml^−1^, AAV Stanford) was injected bilaterally in the DMS (coordinates: +0.5 mm AP, ±1.5 mm ML, −3.5 mm DV).

For the intersectional strategy targeting PnO neurons (Figs. 3, 4 and 6 and Extended Data Figs. 4 and 6), 350 nl of Cre-, FlpO- or Cre/FlpO (con/fon)-dependent retrograde AAV virus^72^ was injected in Gi_left_ in *WT*, *GlyT2*^*GFP*^, *Gad67*^*GFP*^ or *Vglut2*^*Cre*^ mice. The following AAV_retro_ viruses were used for these experiments: AAV_retro_-hSyn1-fDIO-ChrimsonR-tdTom (v413-retro, 4.4 × 10^12^ ml^−1^, Viral Vector Facility, University of Zurich), AAV_retro_-Ef1a-DIO-hChR2(H134R)-EYFP (20298-AAVrg, 1.0 × 10^13^ ml^−1^, Addgene), AAV_retro_-hSyn1-DIO-GtACR2-EGFP (v477-retro, 7.3 × 10^12^ ml^−1^, Viral Vector Facility, University of Zurich), AAV_retro_-hSyn1-fDIO-EGFP-2A-TeLC (v450-retro, 7.7 × 10^12^ ml^−1^, Viral Vector Facility, University of Zurich) or AAV_retro_-hSyn1-con/fon-hChR2(H134R)-EYFP (55645-AAVrg, 7.0 × 10^12^ ml^−1^, Addgene). The injection of AAV_retro_ in Gi was followed 1 week later by injection of 100 nl of AAV1-hSyn1-FlpO (v59-1, 6.7 × 10^12^ ml^−1^, Viral Vector Facility, University of Zurich) or AAV1-hSyn1-Cre (v223-1, 5.2 × 10^12^ ml^−1^, Viral Vector Facility, University of Zurich) in SNr_right_ (coordinates: −3.2 mm AP, +1.20 mm ML, −4.9 mm DV). Injections in SNr were performed at an angle of −15° in the coronal plane to avoid the SC.

For the intersectional strategy targeting *Chx10* Gi neurons (Extended Data Fig. 4), *Chx10*^*Cre*^ mice were injected with 400 nl of AAV5-hSyn1-con/fon-hChR2(H134R)-EYFP (55645-AAV5, 7.0 × 10^12^ ml^−1^, Addgene) in Gi_left_ (coordinates: −6.0 mm AP, −0.8 mm ML, −5.5 mm DV) followed 1 week later by injection of 80 nl of AAV1-hSyn1-FlpO (v59-1, 6.7 × 10^12^ ml^−1^, Viral Vector Facility, University of Zurich) in PnO_right_ (coordinates: −4.3 mm AP, +0.80 mm ML, −4.8 mm DV).

For experiments targeting SNr (Fig. 5 and Extended Data Fig. 5), 100 nl of Cre-dependent AAV virus was injected in SNr_right_ (coordinates: −3.2 mm AP, +1.15 mm ML, −4.9 mm DV) of *Vgat*^*Cre*^ mice. The following viruses were used: AAV5-hSyn1-DIO-GtACR2-EGFP (v477-5, 7 × 10^12^ ml^−1^, Viral Vector Facility, University of Zurich) or AAVdj-Ef1a-DIO-hChR2(E123T/T159C)-p2A-mCherry-WPRE (GVVC-AAV-33, 9 × 10^12^ ml^−1^, AAV Stanford). For retrograde experiments targeting SNr from PnO or SC, 80 nl of AAV_retro_ virus was injected in PnO_right_ (coordinates: −4.3 mm AP, +0.80 mm ML, −4.8 mm DV) or SC_right_ (coordinates: −4.25 mm AP, +1.10 mm ML, −1.8 mm DV) in *Vgat*^*Cre*^ mice. The following viruses were used: AAV_retro_-hSyn1-DIO-GtACR2-EGFP (v477-retro, 7.3 × 10^12^ ml^−1^, Viral Vector Facility, University of Zurich) or AAV_retro_-Ef1a-DIO-hChR2(H134R)-EYFP (20298-AAVrg, 1 × 10^13^ ml^−1^, Addgene).

For chemogenetic activation of *Chx10* Gi neurons (Fig. 6 and Extended Data Fig. 6), 400 nl of AAV5-hSyn1-DIO-hM3D(Gq)-mCherry-WPRE (v89-5, 6 × 10^12^ ml^−1^, Viral Vector Facility, University of Zurich) was injected into Gi_left_ (coordinates: −6.0 mm AP, −0.8 mm ML, −5.5 mm DV) in *Chx10*^*Cre*^ mice.

### Endoscopic Ca^2+^ imaging

Similar surgical procedures as above (‘Stereotaxic viral injections’) were used for gradient index (GRIN) lens implantation. Integrated GRIN lenses (7.3-mm length, 0.6-mm diameter, Inscopix) were modified to image in the Gi. Flexible piano wire (100-µm diameter, SMWL-004-01, Precision Fiber Optics) was cut to 5-mm length. Loctite adhesive was used to attach two 5-mm wires to the GRIN lens at 180° separation, leaving 0.5 mm of wire protruding from the brain-directed surface of the lens. The protruding wire thus stabilized the underlying brain tissue, which helped to reduce motion artifacts during image acquisition. GRIN lens implantation in Gi was performed following bilateral optogenetic fiber implantation in striatum (below, ‘Optogenetics’). Optogenetic fibers were implanted at ±15° in the coronal plane, preserving access to bregma for reference with a pre-track needle and naked GRIN lens. To make space for the GRIN lens, a tissue track was created with a 23-gauge needle advanced toward Gi at a rate of 0.01 mm s^−1^. The pre-track needle was withdrawn upon reaching a DV position of −5.0 mm. A wire-attached GRIN lens was then driven to Gi at a rate of 0.01 mm s^−1^, with final coordinates of −6.0 mm AP, −0.35 mm ML, −5.3 mm DV. The integrated GRIN lens/baseplate was then cemented to the skull using Super Bond C&B (7100, Sun Medical). To reduce ambient light, the Super Bond C&B cement powder was mixed with black carbon powder at a ratio of 10:1.

Endoscopic imaging was performed with an nVoke 2.0 miniature microscope (Inscopix), which was mounted on the integrated GRIN lens/baseplate. A commutator system (Inscopix) was used to minimize cable entanglement. Imaging was performed >3 weeks after implantation following tissue debridement and substantial clearance of the field of view. Electronic focusing was used to determine the optimal field of view, which was maintained for individual mice across imaging sessions. Images were acquired at a rate of 20 Hz with 30–50% light-emitting diode (LED) power, and 2–4× gain. Inscopix data were synchronized with behavioral videos via a tansistor–transistor logic (TTL) pulse passed to the Inscopix DAQ box by Ethovision XT (v.15.0, Noldus). Recordings varied in length between 1 min and 10 min. While the procedures for endoscopic imaging were similar to those in ref. ^56^, we obtained a much smaller yield, and in many animals (not included in this study) we observed no dynamic cells. We have no explanation for these differences. To facilitate future replication and comparisons with the Gi recordings presented herein, we adopted a quantitative definition of cells: bona fide cells were defined as regions of interest (ROIs) that exhibited greater than 3.5% Δ*F/F* over 3 min of recording. Δ*F/F* ranged between 4.0% and 17.0%, with an average Δ*F/F* of 7.4 ± 1.3% (*n* = 7 cells).

### Fiber photometry

Similar surgical procedures as above (‘Stereotaxic viral injections’) were used for fiberoptic cannula implantation. Custom borosilicate fiberoptic cannulas were obtained from Doric Lenses (400-µm diameter, 0.66 numerical aperture, MFC_400/430-0.66_3.0-6.0mm_MF1.25_FLT), attached to a stereotaxic cannula holder (Doric Lenses, SCH_1.25) and driven to the target brain region at a rate of 0.1 mm s^−1^. For optic fiber placement, the skull was prepared using a two-component adhesive (Optibond FL, Kerr). Dental cement (Tetric EvoFlow Bulk Fill, Ivoclar Vivadent) was applied around the ferrule, and cured with ultraviolet light (Superlite 1300, M+W Dental) to affix the implant to the surface of the skull. The cannula holder was then loosened and withdrawn, and individual 6-0 sutures were used to close the skin surrounding the implant. The following fiber coordinates were used: Gi_left_, −6.0 mm AP, −0.35 mm ML, −5.3 mm DV.

Fiber photometry was performed using an RZ10X processor (Tucker-Davis Technologies). The 465-nm (for GCaMP excitation) and 405-nm (for isosbestic excitation) LEDs were driven at modulated frequencies, where isosbestic excitation served as an internal control for photobleaching and movement artifacts. LEDs were driven at a power of 10 mA (peak-to-peak, with 5-mA DC offset), and modulated at 330 Hz (465 nm) and 210 Hz (405 nm). LEDs were coupled to a fluorescence minicube (FMC4_IE(400-410)_E(460-490)_F(500-550)_S, Doric Lenses), which passed 405/465-nm excitation light to the subject via a patch cord (Tucker-Davis Technologies), rotary joint (FRJ_1x1_PT_400-0.57_1m_FCM_0.15m_FCM, Doric Lenses) and subject cable (MFP_400/430/1100-0.57_1m_FCM-MF1.25_LAF, Doric Lenses). Emitted light was filtered via the minicube (500–550 nm) and passed back to an integrated photosensor on the RZ10X processor. Data were acquired in Synapse (v.51891, Tucker-Davis Technologies), which demodulated the 405- and 465-nm signals. Synapse data were synchronized with behavioral videos via a TTL pulse passed to the RZ10X processor by Ethovision XT. Recordings varied in length between 1 min and 10 min.

### Optogenetics

Silica optic fiber (200-µm core, 0.22 numerical aperture, FG200UEA, Thorlabs) was cut to 5 cm, and a micro stripper (T10S13, Thorlabs) was used to strip the acrylate coating on 1.5 cm of the fiber used for mating with a ferrule. Ceramic ferrules (6.7 mm, CFLC230-10, Thorlabs) were then placed on the stripped portion of the optic fiber, and secured using epoxy (F112, Thorlabs). After the epoxy was allowed to dry for 7 d, the stripped fiber was trimmed to a length of 1 mm from the upper surface of the ferrule using an optic fiber scribe (S90R, Thorlabs). A coarse piece of polishing paper (LF5P, Thorlabs) was then used to reduce the protruding fiber to the upper surface of the ferrule on a glass polishing plate (CTG913, Thorlabs), and a finer series of paper (LF3P, LF1P, LF03P, Thorlabs) was used to polish the optic fiber. The fiberoptic scribe was then used to cut the remaining optic fiber with acrylate coating to a length approximately 0.5 mm longer than the final target depth (which varied depending on the target brain region). Similar surgical procedures as above (‘Stereotaxic viral injections’ and ‘Fiber photometry’) were used for optic fiber implantation. The following fiber coordinates were used: Gi, −6.0 mm AP, −0.35 mm ML, −5.1 mm DV; PnO, −4.3 mm AP, +0.8 mm ML, −4.3 mm DV; SNr, −3.2 mm AP, +1.15 mm ML, −4.3 mm DV; striatum, +0.5 mm AP, ±1.5 mm ML, −2.8 mm DV.

For delivery of light pulses, Ethovision XT was used to trigger a Master-8 pulse generator (AMPI) via a TTL pulse. For ChR2 experiments, the Master-8 was used to generate a 1-s train. Experiments in Figs. 1 and 2 and Extended Data Figs. 2–4 utilized a 40-Hz train (15-ms pulse width, 10-ms interval between pulses). For experiments in Figs. 4 and 6 and Extended Data Fig. 6, stimulation frequency was varied between 5 Hz and 40 Hz while keeping laser power and pulse width constant: 5 Hz (15-ms pulse width, 185-ms interval), 10 Hz (15-ms pulse width, 85-ms interval), 20 Hz (15-ms pulse width, 35-ms interval), 40 Hz (15-ms pulse width, 10-ms interval). For GtACR2 experiments, the Master-8 was used to generate a 1-s continuous pulse. The Master-8 triggered a 473-nm laser (Laserglow Technologies), which delivered light via a patch cable (M74L01, Thorlabs), rotary joint (RJ1, Thorlabs) and optic fiber cable (FG105UCA, Thorlabs) to the optic fiber–ferrule implant. Pseudo-random optogenetic stimulation was triggered while mice were moving through the center of the arena. In two of nine mice with GtACR2 expression in PnO-Vglut2_contra_ neurons (Fig. 4i–k), GtACR2 initiated a short-lasting left (contralateral) turn immediately followed by a prolonged right (ipsilateral) turn. This behavior is consistent with early spiking caused by GtACR2 followed by prolonged neuronal inhibition^73^.

### Open-field behavior

Open-field behavioral analysis was performed in custom-fabricated 50 × 50-cm^2^ arenas. Testing in the spiral maze (Fig. 4l–n) was carried out as described previously^23^. Briefly, mice were placed in the center of the maze, and allowed to explore the maze to completion or until 10 min had elapsed. For optogenetics in 6-OHDA-lesioned mice (Fig. 6 and Extended Data Fig. 6), mice were tested in a 15-cm-diameter cylinder, which promotes locomotor turning^23^. Behavior was captured using an overhead camera (25 frames per second, 1,280 × 960-square pixels, acA1300–60gm camera, Basler; H3Z4512CS-IR lens, Computar), and recorded using Ethovision XT. For chemogenetics in 6-OHDA-lesioned mice (Fig. 6 and Extended Data Fig. 6), open-field behavior was captured in four 50 × 50-cm^2^ arenas imaged simultaneously using a bottom-view camera (30 frames per second, GO-5000M-USB camera, JAI; LM12HC lens, Kowa Optical Products), and recorded in eBUS player (Pleora Technologies).

### 6-OHDA lesions

At 30 min before unilateral 6-OHDA injection, mice received an intraperitoneal injection of desipramine (25 mg kg^−1^) to prevent 6-OHDA damage to the noradrenergic system. We injected 1 µl of 6-OHDA (5 mg ml^−1^ dissolved in a 0.2% ascorbic acid/saline solution) unilaterally into the right DMS as above (‘Stereotaxic viral injections’). The injection was performed at the following coordinates: +0.5 mm AP, +1.5 mm ML, −3.5 mm DV.

To improve recovery after 6-OHDA lesion, supplemental nutrition including wet chow pellets, condensed milk and Nutella was given daily. Supplemental nutrition was provided 1 week before 6-OHDA lesion to prevent food neophobia and was discontinued upon recovery from the lesion. 6-OHDA-lesioned mice were assessed daily for weight loss and dehydration within the first 10 d post-lesion, and thereafter every 2 d. Dehydrated mice received fluids (either saline or 5% glucose), and hypothermic mice were warmed by placing their cage on a heating pad. Mice were euthanized if they exhibited greater than 15% weight loss.

### Chemogenetics

CNO (4936, Tocris) was dissolved in 0.9% saline at a concentration of 0.1 mg ml^−1^. Saline or CNO (1 mg kg^−1^) was then administered intraperitoneally, and mice were placed in an open-field arena. Open-field behavior was recorded for 40 min.

### Immunochemistry and in situ hybridization

For immunochemistry, mice were administered an anesthetic overdose of pentobarbital (250 mg kg^−1^). Transcardial perfusion was subsequently performed with 4 °C PBS followed by 4% paraformaldehyde in 0.1 M phosphate buffer (HL96753.1000, HistoLab). Brain tissue was dissected free and post-fixed for 3 h in 4% paraformaldehyde at 4 °C. The tissue was transferred to 27.5% sucrose dissolved in PBS for cryoprotection and incubated at 4 °C for 24–48 h. Tissue was washed with PBS to remove excess sucrose, patted dry and embedded in NEG-50 medium (D22267, ThermoFisher Scientific). Tissue blocks were then frozen on dry ice and stored at −20 °C. Frozen coronal or sagittal sections (30–50 µm) were obtained on a cryostat (CryoStar NX70, ThermoScientific), and mounted on slides (Superfrost Plus, ThermoScientific).

Sections were rehydrated in PBS with 0.5% Triton-X100 (PBS-T; X100, Sigma Aldrich) for 5 min, and then blocked with PBS-T + 10% normal donkey serum (Jackson ImmunoResearch) for 2 h at room temperature. Sections were incubated in primary antibodies diluted in blocking solution overnight at 4 °C. The following primary antibodies were used: rabbit anti-dsRed/mCherry/tdTomato (1:1,000, 632496, Takahara Bio), chicken anti-GFP (1:1,000, ab13970, Abcam), rabbit anti-TH (1:1,500, AB152, Millipore). Slides were washed 4 × 10 min in PBS-T and incubated with goat anti-chicken Alexa Fluor 488 (1:500, A11039, ThermoFisher Scientific) and/or donkey anti-rabbit Alexa Fluor 568 (1:500, A10042, ThermoFisher Scientific) secondary antibodies diluted in blocking solution. Counterstaining was performed with Hoechst 33342 (1:2,000, 62249, ThermoFisher Scientific) or NeuroTrace 435 (1:200, N21479, ThermoFisher Scientific). Coverslips were mounted using mowiol 4–88 mounting medium (475904-M, Sigma Aldrich). Images were acquired using a Zeiss LSM 900.

For in situ hybridization, mice were administered an anesthetic overdose of pentobarbital, and transcardial perfusion was performed with 4 °C PBS. Brain tissue was rapidly dissected free and flash-frozen in isopentane cooled on dry ice. Tissue was then embedded in NEG-50 medium and stored at −80 °C before cutting. Cryosections (14 µm) were mounted and stored at −80 °C before hybridization. In situ hybridization for target genes was performed using the RNAscope Multiplex Fluorescent Assay v2 (323110, Advanced Cell Diagnostics) using the following RNAscope Target Probes (Advanced Cell Diagnostics): *tdTom* (317041-C3), *Slc17a6* (*Vglut2*, 319171-C2), *Slc32a1* (*Vgat*, 319191-C1). Individual channels were developed using Opal 520, 570 and 690 dyes (1:1,500, Akoya Biosciences). Sections were counterstained with DAPI, and coverslips were mounted with ProLong Diamond Antifade (P36961, ThermoFisher Scientific) medium. Images were acquired in ZEN software (v.3.3.89, Carl Zeiss Microscopy) using an LSM 900 confocal microscope (Carl Zeiss Microscopy).

### Behavioral tracking

Behavioral tracking was performed offline using DeepLabCut (https://github.com/DeepLabCut/)^74,75^. DeepLabCut v.2.0 was installed on a PC equipped with a GEforce RTX2080 graphics card. Videos were downsampled to a resolution of 640 × 480-square pixels before analysis. An initial training dataset was assembled from approximately 400 frames extracted from videos representing different mice, behavioral sessions and behavioral paradigms; frames were extracted from videos of mice with no cable, an optogenetic cable, a miniscope, a miniscope and optogenetic cable, a fiber photometry cable and both a fiber photometry and optogenetic cable. Frames in the training dataset were manually labeled for the tip of the nose, neck, body center, tail base and tail tip. A resnet50 network was trained for 1 × 10^6^ iterations on the initial training dataset. Videos representative of different behavioral paradigms were then analyzed using the restnet50 network, and outlier frames were extracted. Outlier frames were scored and merged with the initial training dataset to assemble a modified training dataset. A resnet50 network was generated using the modified training dataset, and this network was used for subsequent analyses. For chemogenetic experiments (Fig. 6 and Extended Data Fig. 6), behavioral tracking was performed offline in Ethovision XT, which extracted nose, body center and tail base position.

### Kinematics

The following kinematic variables were derived using custom scripts in MATLAB (v.R2021a, MathWorks) from $x,{y}$ position data generated by DeepLabCut: direction, angular velocity, speed and turn radius. For Figs. 1, 2 and 6 and Extended Data Figs. 2 and 6, direction was calculated from the body–snout vector. For Figs. 4 and 5 and Extended Data Figs. 3–5, direction was calculated separately for the body (body–neck vector) and the head relative to the body (head; direction of the neck–snout vector − direction of the body vector):

$$\theta ={\tan }^{-1}\frac{{y}_{2}-{y}_{1}}{{x}_{2}-{x}_{1}}$$

where $\theta$ is direction in degrees. Phase unwrapping of the *θ*_*t*_ time series was used to represent changes in direction continuously through a four-quadrant space. For Fig. 4e, each trial was adjusted such that direction was equal to 0 at light onset. Angular velocity data were then calculated from *θ*_*t*_, with a bin of 80 ms (representing 3 frames):

$${av}_{t}=\frac{{\theta }_{t+1}-{\theta }_{t-1}}{0.08}$$

where ${av}$ is the angular velocity in ° s^−1^. Positive angular velocity represents counterclockwise (left) movement, and negative angular velocity represents clockwise (right) movement. Speed was calculated from the body position, converting pixel size to cm, with a bin of 80 ms:

$${v}_{t}=\frac{\sqrt{{({x}_{t+1}-{x}_{t-1})}^{2}+{({\,y}_{t+1}-{y}_{t-1})}^{2}}}{0.08}$$

where $v$ is the speed in cm s^−1^. Turning radius was approximated from ${v}_{t}$ and ${av}_{t}$, converting angular velocity to rad s^−1^:

$${r}_{t}=\frac{{v}_{t}}{{av}_{t}}$$

where $r$ is the turning radius in cm.

For Fig. 6d–f and Extended Data Fig. 6b–f, revolution analysis was performed in Ethovision XT, where 360° left (counterclockwise) and right (clockwise) revolutions were quantified using the tail base to body center vector, with a 50° threshold for switching between measurement of left and right revolutions. For continuous measurement of leftward movement preference (Fig. 6e and Extended Data Fig. 6b,e), left revolutions were quantified as a percentage of the total number of revolutions in 5-min bins. If no revolutions were recorded during a 5-min bin, left preference was reported as 50%. For box-and-whisker plots (Fig. 6d,f and Extended Data Fig. 6c,d,f), revolutions were quantified from 10–40 min after injection of saline or CNO.

### Ca^2+^ analysis

Post-processing of endoscopic imaging data was performed in Inscopix Data Processing software (IDPS v.1.6.0, Inscopix). Inscopix (.isdx) files were imported into IDPS and underwent preprocessing to crop the field of view and downsample spatial resolution (2×). Spatial filtering was performed with low- (0.005 pixels^−1^) and high- (0.5 pixels^−1^) pass Gaussian filters, and motion correction was performed using a ROI that contained the imaging field. Δ*F/F* was defined relative to the mean frame (*F*_0_). Manual ROIs were used to register putative cells, and bona fide cells were defined as ROIs that exhibited greater than 3.5% Δ*F/F*. Δ*F/F* ranged between 4.0% and 17.0%, with an average Δ*F/F* of 7.4 ± 1.3% (*n* = 7 cells).

Post-processing of fiber photometry data was performed in Spike2 (v.7.17, Cambridge Electronic Design). Synapse files (.TSQ) were imported into Spike2, and the fluorescence signal (*F*_*t*_) was defined as the difference between the 465-nm and 405-nm channels (GCaMP excitation − isosbestic excitation). The signal was smoothed using a low-pass digital finite impulse response filter and linearly downsampled to 25 Hz. Δ*F/F* was defined relative to the mean fluorescence of the trace (*F*_0_); Δ*F/F* was defined as (*F*_*t*_ − *F*_0_)/*F*_0_. GCaMP7s signal quality was evaluated, and animals that exhibited greater than 10% Δ*F/F* were selected for further analysis. Δ*F/F* ranged between 13% and 73%, with an average Δ*F/F* of 41 ± 5% (*n* = 14 mice).

For analysis of Δ*F/F* activity during spontaneous left or right turns and ChR2-stimulation trials (Figs. 1d,e and 2c,d and Extended Data Fig. 2b), row Δ*F/F Z*-score was calculated for each event/trial to scale for the difference in magnitude of Δ*F/F* activity exhibited across cells (for endoscopic imaging) or animals (for fiber photometry). Row *Z*-scores were calculated based on the mean and standard deviation of Δ*F/F* values during the baseline period (time −2.5 to 0 s).

### Cross-correlation

To evaluate the correlation between GCaMP fluorescence and behavior (Fig. 1c), we performed cross-correlation between the angular velocity ($av$) and the derivative of the Δ*F/F* (ΔΔ*F/F*) time series^76^. Cross-correlation was performed using a custom MATLAB script:

$$\left[{\mathrm{CC}},{\rm{lags}}\right]={\rm{crosscorr}}({av},{{\Delta}{\Delta}F/F},400)$$

where CC is the cross-correlation calculated with a maximum lag of 400 frames (20 s). Cross-correlation analysis was performed using 3 min of recording in the absence of ChR2 stimulation.

### Segmentation of spontaneous turns

Spontaneous left turns, straight events and right turns (Fig. 1d,e and Supplementary Video 2) were segmented from ${{av}}_{t}$ using a custom MATLAB script. Left turns were defined as an angular velocity greater than 200° s^−1^ within 1 s of 0° s^−1^. Straight events were defined as an angular velocity that remained at ±20° s^−1^ for 1 s. Right turns were defined as an angular velocity less than −200° s^−1^ within 1 s of 0° s^−1^. Event onset was considered 0° s^−1^. For endoscopic imaging, the first 20 left turns, 20 straight events and 20 right turns were quantified for each animal. For fiber photometry, the first ten left turns, ten straight events and ten right turns were quantified for each animal.

### Co-expression

Analysis of neuronal co-expression of *tdTom*, *Vglut2* and *Vgat* in Fig. 3c was carried out in ImageJ^77^ using the ROI 1-Click Tools plugin. Neurons were considered *tdTom*^+^ if they exhibited at least four positive puncta in close association with a DAPI-labeled nucleus. In total, 98.4 ± 0.5% (*n* = 4 mice) of *tdTom*^+^ neurons exhibited co-expression of either *Vglut2* or *Vgat*. Further, 1.6 ± 0.5% of *tdTom*^+^ neurons were not assigned as *tdTom*^*+*^*Vglut2*^*+*^ *or tdTom*^*+*^*Vgat*^*+*^ due to putative co-expression of both *Vglut2* and *Vgat* or a lack of either *Vglut2* or *Vgat* co-expression. Similar analysis was performed to assess co-expression of tdTom and GFP in Fig. 3d.

### Atlas registration

Atlas registration of PnO-Vglut2_contra_ neurons in Fig. 3e was performed by manually assigning sections to corresponding coronal plates in Paxinos and Franklin’s reference atlas^37^. Neuronal position within each image was manually registered using ImageJ, converting pixel size to µm. A landmark reference at 0.0 mm ML, −1.25 mm DV was used to transform coordinates onto the reference atlas. Sections were quantified every 100 µm through the rostrocaudal extent of PnO, with 11–14 sections quantified for each mouse. An average of 188 ± 34 neurons were registered for each mouse (*n* = 3 mice).

### Mouse cohorts

Sample sizes are similar to those reported previously^22,23,78,79^; no formal statistical methods were used to pre-determine sample size. A block design was used to randomly allocate mice to different groups, with an effort to include both males and females in each group (sex for each experiment is reported in Supplementary Table 1). Sex-specific responses were examined post hoc for those experiments with equivalent numbers of males and females; however, no evidence for sexually dimorphic responses was uncovered.

### Blinding

Data collection and analysis were not blinded to the experimenter; however, data collection and analysis were automated to limit the influence of the experimenter on outcome.

### Exclusion criteria

For endoscopic imaging (Figs. 1 and 2), ROIs that exhibited less than 3.5% Δ*F/F* were excluded from analysis. Additionally, mice were excluded on the basis of lens movement artifact, lack of cells or poor field of view. For fiber photometry (Figs. 1 and 2 and Extended Data Fig. 2), mice that exhibited less than 10% Δ*F/F* were excluded from analysis. Additionally, mice were excluded based on the stability of the isosbestic control signal; an unstable isosbestic signal is indicative of fiber movement artifact. For optogenetics (Figs. 2 and 4–6 and Extended Data Figs. 2–6), mice were excluded from analysis if the fiber position or viral infection was off target. For chemogenetics (Fig. 6 and Extended Data Fig. 6), mice were excluded from analysis if the viral infection exhibited substantial spread across the midline. For 6-OHDA lesions (Fig. 6 and Extended Data Fig. 6), mice were euthanized if they exhibited greater than 15% weight loss (representing the pre-defined humane endpoint). 6-OHDA lesion extent was evaluated via TH staining of the SNc at day 20 after injection of 6-OHDA, following testing in the chronic stage. Mice were excluded from analysis if the lesion exhibited less than 70% efficacy (the lesioned SNc exhibited greater than 30% of neurons relative to the intact side). For optogenetics, chemogenetics and 6-OHDA lesions, exclusions were performed post-experimentally upon examination of the tissue.

### Significance

A significant cross-correlation between angular velocity and the derivative of the Δ*F/F* (Fig. 1c) was defined as a peak within 0.5-s lag greater than (cross-correlated) or less than (anti-cross-correlated) 2 s.d. of a normally distributed population mean:

$$\mu \pm \frac{2}{\sqrt{n-{\rm{|}}k{\rm{|}}}}$$

where $\mu$ is the mean (zero), $n$ is the number of observations and $k$ is the lag. For pairwise comparisons (Figs. 2f, 3c,d and 4n and Extended Data Fig. 2c), a two-tailed paired *t*-test was used to determine significance. For multiple comparisons, a one-way (Figs. 1f, 2e, 4d,e,h,k, 5d and 6i and Extended Data Figs. 2c, 3b,c, 4b,d, 5a–c and 6i) or two-way (Fig. 6d,f and Extended Data Fig. 6c,d,f) analysis of variance (ANOVA)—with repeated measures where appropriate—was used to determine whether significance was present. If significance was present, *P* values were assigned in multiple comparisons testing using Tukey’s post hoc test. Data were assumed to exhibit normal distribution, but this was not tested. *P* < 0.05 was considered statistically significant, with *P < 0.05, **P < 0.01 and ***P < 0.001. *P* values and *n* values are reported in the figure legends. Full details on statistical analyses, including test statistics and cohort composition, are reported in Supplementary Table 1. Statistics were performed in GraphPad Prism 9.3.1.

### Plots

Time series graphs, heat maps, violin plots, box-and-whisker plots and bar graphs were generated in GraphPad Prism v.9.3.1. Neuronal density plots in Fig. 3e were generated using a custom script in RStudio. Time series data for angular velocity, direction and Δ*F/F Z*-score represent mean ± s.e.m. Violin plots give the median, the 25th and 75th percentiles and the range. Box-and-whisker plots represent the median, 25th and 75th percentiles and range. Individual data points (*n*) are plotted for each comparison, where *n* values represent distinct mice—except for where indicated otherwise (Figs. 1d and 2d and Extended Data Fig. 3b,c). Figures were prepared in Adobe Illustrator, and videos were prepared in Adobe Premier Pro. Time series graphs in Supplementary Videos 1–8 were generated using custom MATLAB scripts. Experimental paradigms were illustrated using plates derived from the Allen Mouse Brain Atlas^80^.

### Reporting summary

Further information on research design is available in the Nature Portfolio Reporting Summary linked to this article.

## Online content

Any methods, additional references, Nature Portfolio reporting summaries, source data, extended data, supplementary information, acknowledgements, peer review information; details of author contributions and competing interests; and statements of data and code availability are available at 10.1038/s41593-024-01569-8.

## Supplementary information

Reporting Summary

Supplementary Table 1 Statistics summary. The table reports statistical methods employed, test statistics and cohort composition for each dataset.

Supplementary Video 1 Endoscopic Ca^2+^ imaging of *Chx10* Gi neurons. Related to Fig. 1.

Supplementary Video 2 Examples of spontaneous left turns, straight events and right turns segmented from angular velocity time series. Related to Fig. 1.

Supplementary Video 3 Examples of turns evoked via D1- or D2-SPN stimulation. Related to Fig. 2 and Extended Data Figs. 2 and 3.

Supplementary Video 4 Optogenetic stimulation or inhibition of PnO-Vglut2_contra_ neurons. Related to Fig. 4.

Supplementary Video 5 Tight control of turning kinematics via frequency-dependent modulation of PnO-Vglut2_contra_ neurons. Related to Fig. 4.

Supplementary Video 6 Optogenetic inhibition of Vgat^+^ SNr neurons. Related to Fig. 5.

Supplementary Video 7 Chemogenetic activation of *Chx10* Gi neurons in mice with acute unilateral striatal dopamine depletion. Related to Fig. 6. Representative examples are taken from early (0–10 min), intermediate (10–20 min) or late stages (>20 min) following administration of CNO.

Supplementary Video 8 Optogenetic activation of PnO-Vglut2_contra_ neurons in mice with acute unilateral striatal dopamine depletion. Related to Fig. 6.

## Data Availability

Preprocessed behavioral videos, DeepLabCut tracking files and labeled videos associated with spontaneous turns (Fig. 1) and optogenetic experiments (Figs. 2 and 4–6 and Extended Data Figs. 2–6) are available at 10.17894/ucph.b2081dd4-9ae8-4dfa-bc75-b4cbd197b879. Any other data or materials are available from the corresponding authors upon request.

## Code availability

The code used to analyze data and produce figure content associated with kinematic analysis of spontaneous turns (Fig. 1) and optogenetic experiments (Figs. 2 and 4–6 and Extended Data Figs. 2–6) is available at 10.17894/ucph.b2081dd4-9ae8-4dfa-bc75-b4cbd197b879. Portions of this code were generated with assistance from ChatGPT (GPT-4) by OpenAI. Any other code is available from the corresponding authors upon request.
