# Supplementary material for: Basal ganglia–spinal cord pathway that commands locomotor gait asymmetries in mice
Source: Nat Neurosci. 2024 Feb 12;27(4):716–27. doi: 10.1038/s41593-024-01569-8 (PMC11001584; doi:10.1038/s41593-024-01569-8)
Supplement: Supplementary file 1 — Reporting Summary [file 41593_2024_1569_MOESM1_ESM.pdf]

Corresponding author(s): Jared M. Cregg, Ole Kiehn

Last updated by author(s): Jan 4, 2024

# Reporting Summary

Nature Portfolio wishes to improve the reproducibility of the work that we publish. This form provides structure for consistency and transparency in reporting. For further information on Nature Portfolio policies, see our Editorial Policies and the Editorial Policy Checklist.

## Statistics

For all statistical analyses, confirm that the following items are present in the figure legend, table legend, main text, or Methods section.

| n/a | Confirmed | |
|---|---|---|
| ☐ | ☒ | The exact sample size (*n*) for each experimental group/condition, given as a discrete number and unit of measurement |
| ☐ | ☒ | A statement on whether measurements were taken from distinct samples or whether the same sample was measured repeatedly |
| ☐ | ☒ | The statistical test(s) used AND whether they are one- or two-sided<br>*Only common tests should be described solely by name; describe more complex techniques in the Methods section.* |
| ☒ | ☐ | A description of all covariates tested |
| ☐ | ☒ | A description of any assumptions or corrections, such as tests of normality and adjustment for multiple comparisons |
| ☐ | ☒ | A full description of the statistical parameters including central tendency (e.g. means) or other basic estimates (e.g. regression coefficient) AND variation (e.g. standard deviation) or associated estimates of uncertainty (e.g. confidence intervals) |
| ☐ | ☒ | For null hypothesis testing, the test statistic (e.g. *F*, *t*, *r*) with confidence intervals, effect sizes, degrees of freedom and *P* value noted<br>*Give P values as exact values whenever suitable.* |
| ☒ | ☐ | For Bayesian analysis, information on the choice of priors and Markov chain Monte Carlo settings |
| ☒ | ☐ | For hierarchical and complex designs, identification of the appropriate level for tests and full reporting of outcomes |
| ☒ | ☐ | Estimates of effect sizes (e.g. Cohen's *d*, Pearson's *r*), indicating how they were calculated |

*Our web collection on statistics for biologists contains articles on many of the points above.*

## Software and code

Policy information about availability of computer code

| | |
|---|---|
| Data collection | Ethovision XT (v15.0, Noldus) was used to capture behavioral videos. Fiber photometry data was acquired in Synapse software (version 51891, Tucker-Davis Technologies). Endoscopic Ca2+ imaging data was acquired using Inscopix Data Acquisition software (Inscopix). Fluorescent images were captured using ZEN software (version 3.3.89, Carl Zeiss Microscopy). |
| Data analysis | DeepLabCut (v2.0) or EthovisionXT (v15.0, Noldus) were used to carry out tracking analysis. Kinematic variables were derived using custom scripts in MATLAB (MathWorks, R2021a). Spike2 (version 7.17, Cambridge Electronic Design) was used for post-processing of fiber photometry data, with further analysis using custom MATLAB scripts. Inscopix Data Processing software (IDPS v1.6.0, Inscopix) was used for post-processing of endoscopic Ca2+ imaging data, with further analysis using custom MATLAB scripts. Statistics were performed in GraphPad Prism 9.3.1. Time series graphs, heat maps, violin plots, and box-and-whisker plots, and bar graphs were generated in GraphPad Prism 9.3.1.<br><br>The code used to analyze data and produce figure content associated with kinematic analysis of spontaneous turns (Fig. 1) and optogenetic experiments (Figs. 2, 4-6, Extended Data Figs. 2-6) are available at https://doi.org/10.17894/ucph.b2081dd4-9ae8-4dfa-bc75-b4cbd197b879. Portions of this code were generated with assistance from ChatGPT (GPT-4) by OpenAI. Any other code is available from the corresponding authors upon request. |

For manuscripts utilizing custom algorithms or software that are central to the research but not yet described in published literature, software must be made available to editors and reviewers. We strongly encourage code deposition in a community repository (e.g. GitHub). See the Nature Portfolio guidelines for submitting code & software for further information.

## Data

Policy information about availability of data

All manuscripts must include a data availability statement. This statement should provide the following information, where applicable:

- Accession codes, unique identifiers, or web links for publicly available datasets
- A description of any restrictions on data availability
- For clinical datasets or third party data, please ensure that the statement adheres to our policy

Preprocessed behavioral videos, DeepLabCut tracking files, and labeled videos associated with spontaneous turns (Fig. 1) and optogenetic experiments (Figs. 2, 4-6, Extended Data Figs. 2-6) are available at https://doi.org/10.17894/ucph.b2081dd4-9ae8-4dfa-bc75-b4cbd197b879. Any other data or materials are available from the corresponding authors upon request.

## Human research participants

Policy information about studies involving human research participants and Sex and Gender in Research.

| | |
|---|---|
| Reporting on sex and gender | N/a |
| Population characteristics | N/a |
| Recruitment | N/a |
| Ethics oversight | N/a |

Note that full information on the approval of the study protocol must also be provided in the manuscript.

# Field-specific reporting

Please select the one below that is the best fit for your research. If you are not sure, read the appropriate sections before making your selection.

☒ Life sciences ☐ Behavioural & social sciences ☐ Ecological, evolutionary & environmental sciences

For a reference copy of the document with all sections, see nature.com/documents/nr-reporting-summary-flat.pdf

# Life sciences study design

All studies must disclose on these points even when the disclosure is negative.

| | |
|---|---|
| Sample size | Sample sizes are similar to those reported previously (Cregg et al, Nat Neurosci, 2020; Caggiano et al, Nature 2018; Jennings et al, Nature, 2019; Evans et al, Nature 2018); no formal statistical methods were used to pre-determine sample size. |
| Data exclusions | For endoscopic imaging (Figs. 1, 2), ROIs which exhibited less than 3.5% dF/F were excluded from analysis. Additionally, mice were excluded on the basis of lens movement artifact, lack of cells, or poor field of view. For fiber photometry (Figs. 1, 2, Extended Data Fig. 2), mice which exhibited less than 10% dF/F were excluded from analysis. Additionally, mice were excluded based on the stability of the isosbestic control signal; an unstable isosbestic signal is indicative of fiber movement artifact. For optogenetics (Figs. 2, 4, 5, 6, Extended Data Figs. 2-6), mice were excluded from analysis if the fiber position or viral infection was off target. For chemogenetics (Fig. 6, Extended Data Fig. 6), mice were excluded from analysis if the viral infection exhibited substantial spread across the midline. For 6-OHDA lesions (Fig. 6, Extended Data Fig. 6), mice were euthanized if they exhibited greater than 15% weight loss (representing the pre-defined humane endpoint). 6-OHDA lesion extent was evaluated via TH staining of the SNc at day 20 after injection of 6-OHDA, following testing in the chronic stage. Mice were excluded from analysis if the lesion exhibited less than 70% efficacy (the lesioned SNc exhibited greater than 30% of neurons relative to the intact side). For optogenetics, chemogenetics, and 6-OHDA lesions, exclusions were performed post-experimentally upon examination of the tissue. |
| Replication | Several trials were performed for each of the experiments. For some experiments, several cohorts (independent experiments) were carried out to obtain the final datasets. In all cases, attempts at replication were successful. |
| Randomization | A block design was used to randomly allocate mice to different groups, with an effort to include both males and females in each group (sex for each experiment is reported in Supplementary Table 1). |
| Blinding | Data collection and analysis were not blinded to the experimenter; however, data collection and analysis were automated to limit the influence of the experimenter on outcome. |

# Reporting for specific materials, systems and methods

We require information from authors about some types of materials, experimental systems and methods used in many studies. Here, indicate whether each material, system or method listed is relevant to your study. If you are not sure if a list item applies to your research, read the appropriate section before selecting a response.

## Materials & experimental systems

| n/a | Involved in the study |
| --- | --- |
| ☐ | ☒ Antibodies |
| ☒ | ☐ Eukaryotic cell lines |
| ☒ | ☐ Palaeontology and archaeology |
| ☐ | ☒ Animals and other organisms |
| ☒ | ☐ Clinical data |
| ☒ | ☐ Dual use research of concern |

## Methods

| n/a | Involved in the study |
| --- | --- |
| ☒ | ☐ ChIP-seq |
| ☒ | ☐ Flow cytometry |
| ☒ | ☐ MRI-based neuroimaging |

# Antibodies

| | |
| --- | --- |
| Antibodies used | The following primary antibodies were used: rabbit anti-dsRed/mCherry/tdTomato (1:1000, 632496, Clontech), chicken anti-GFP (1:1000, ab13970, Abcam), rabbit anti-TH (1:1500, AB152, Millipore). The following secondary antibodies were used: Alexa Fluor 488 goat anti-chicken IgY (1:500, A11039, ThermoFisher Scientific), Alexa Fluor 568 donkey anti-rabbit IgG (1:500, A10042, ThermoFisher Scientific). |
| Validation | Antibodies have been validated with either western blot or immunohistochemistry on mouse samples, as indicated on the manufacturer's product page. Antibodies have also been validated in a number of publications as indicated on the Antibody Registry (AB10013483; AB300798; AB390204; AB2534096; AB2534017). |

# Animals and other research organisms

Policy information about studies involving animals; ARRIVE guidelines recommended for reporting animal research, and Sex and Gender in Research

| | |
| --- | --- |
| Laboratory animals | Experiments were performed in adult mice greater than 8 weeks of age, with an effort to include similar numbers of male and female mice. The following strains were used for the experiments herein: D1RCre (Gensat EY217), D2RCre (Gensat ER44), Chx10Cre (Azim et al, Nature 2014), R26RtdTom (Ai14, Jackson Stock #007914), WT (C57BL6/J, Jackson Stock #000664), GlyT2GFP (Zeilhofer et al, J Comp Neurol, 2005), GAD67GFP (Tamamaki et al, J Comp Neurol, 2003; Restrepo et al, J Comp Neurol, 2009), Vglut2Cre (Borgius et al, Mol Cell Neurosci, 2010), VgatCre (Hagglund et al, PNAS, 2013; Vong et al, Neuron, 2011, Jackson Stock #028862). |
| Wild animals | No wild animals were used. |
| Reporting on sex | Experiments were performed with an effort to include similar numbers of male and female mice (sex for each experiment is reported in Supplementary Table 1). Sex-specific responses were examined post hoc for those experiments with equivalent numbers of males and females; however, no evidence for sexually dimorphic responses was uncovered. |
| Field-collected samples | No field-collected samples were used. |
| Ethics oversight | Animal procedures were performed in accordance with European Union Directive 2010/63/EU, and approved by the Danish Animal Inspectorate (Dyreforsøgstilsynet, permits 2017-15-0201-01172 and 2022-15-0201-01131) as well as the clinical veterinarians at the Department of Experimental Medicine, Faculty of Health and Medical Sciences, University of Copenhagen (plans P19-134, P21-323, P22-502, A20-160, and A23-154). |

Note that full information on the approval of the study protocol must also be provided in the manuscript.
